# Time-Dependent Afterglow from a Single Component Organic Luminogen

**DOI:** 10.34133/2021/9757460

**Published:** 2021-08-27

**Authors:** Tianjia Yang, Yunzhong Wang, Jixuan Duan, Shuangyu Wei, Saixing Tang, Wang Zhang Yuan

**Affiliations:** School of Chemistry and Chemical Engineering, Frontiers Science Center for Transformative Molecules, Shanghai Key Lab of Electrical Insulation and Thermal Aging, Shanghai Jiao Tong University, Shanghai 200240, China

## Abstract

Pure organic luminogens with long-persistent luminescence have been extensively studied, on account of their fundamental research significance and diverse utilizations in anticounterfeiting, bioimaging, encryption, organic light-emitting diodes, chemo-sensing, etc. However, time-dependent color-tunable afterglow is rarely reported, especially for single-component materials. In this work, we reported an organic luminogen with time-dependent afterglow, namely, benzoyleneurea (BEU), with multiple persistent room-temperature phosphorescence (p-RTP) and thermally activated delayed fluorescence (TADF) in single crystals. While the lifetime of TADF is relatively short (~1.2 ms), those for p-RTP are as long as around 369~754 ms. The comparable but different decay rates of diversified p-RTP emissions endow BEU crystals with obvious time-dependent afterglow. The existence of multiple emissions can be reasonably illustrated by the clustering-triggered emission (CTE) mechanism. Single-crystal structure illustrates that the combination of benzene ring and nonconventional chromophores of ureide helps facilitate divergent intermolecular interactions, which contribute to the formation of varying emissive species. Moreover, its methyl- and chloro-substituted derivatives show similar multiple p-RTP emissions. However, no time-dependent afterglows are observed in their crystals, due to the highly approaching lifetimes. The afterglow color variation of BEU crystals grants its applications in advanced anticounterfeiting field and information encryption.

## 1. Introduction

Pure organic persistent room-temperature phosphorescence (p-RTP) has been broadly developed, due to the fundamental significance, as well as great potentials in vast fields including anticounterfeiting [1, 2], encryption [3–6], bioimaging [2, 7], and chemo-sensing [8]. People aim to prolong the lifetime of organic p-RTP and meanwhile endeavor in a high phosphorescence efficiency [9]. Creating sufficient intersystem crossing (ISC) and reducing nonradiative dissipation of triplet excitons are two major principles to obtain remarkable RTP [10]. Considering these ideas, strategies including crystal engineering [11], H-aggregation [3], host-guest complexes [12], and rigid molecular matrix [5, 6] were applied to reduce molecular vibrational dissipations. Moreover, heteroatoms [13], aromatic carbonyls [14], and halogen atoms [15] are introduced to promote spin-orbit coupling (SOC), to yield more triplet excitons. However, the majority of currently reported afterglow materials depict single emission color, achieving two or more colors from single components remains challenging [16–18].

Very recently, luminescent materials with tunable photoluminescence (PL) and/or RTP have attracted much attention because they provide significant information of the excitons at the aggregate level and supply emerging applications in advanced data encryption, anticounterfeiting, UV detection [16–26], etc. For example, Huang and An et al. obtained dual phosphorescent emission from monomers and H-aggregates of triazine derivatives with excitation-dependent color-tunable p-RTP, which renders them applicable in UV detection [18]. Currently, the PL color tunability is mostly achieved by modifying the molecular structures [27], achieving polymorphs [23, 28], changing the temperature or the excitation wavelength (*λ*_ex_) [18, 22, 29, 30], and so on [31–33]. Organics with time-dependent color-tunable afterglow, however, are rarely reported [14, 34], especially for single-component materials. Chi and Zhang et al. obtained time-dependent afterglow emission in a single organic by constructing intermolecular hydrogen-bonding interaction [14]. Yang et al. reported a carbazole-based single-component organic crystal with both TADF and p-RTP emissions, which exhibited time-dependent afterglow by tuning the ground state properties [34]. More strategies on designing molecules with time-dependent afterglows are urgently needed.

Based on previous researches on nonconventional luminophores with *λ*_ex_-dependent color-tunable p-RTP, it is found that sufficient through-space conjugation (TSC), caused by inter/intramolecular interactions of nonconventional luminophores, plays an important role in obtaining multiple emissive species [35–40]. A clustering-triggered emission (CTE) mechanism is then introduced to explain the phenomenon [41–43]. Therefore, it is natural to conceive that the introduction of electron-rich nonconventional chromophores into a small aromatic unit may also facilitate the formation of multiple emissive centers in a single component. By adjusting the different emissive species with various emission maxima (*λ*_em_*s*), relative intensities, and lifetimes, it might be possible to achieve time-dependent afterglows.

**Figure 1:**
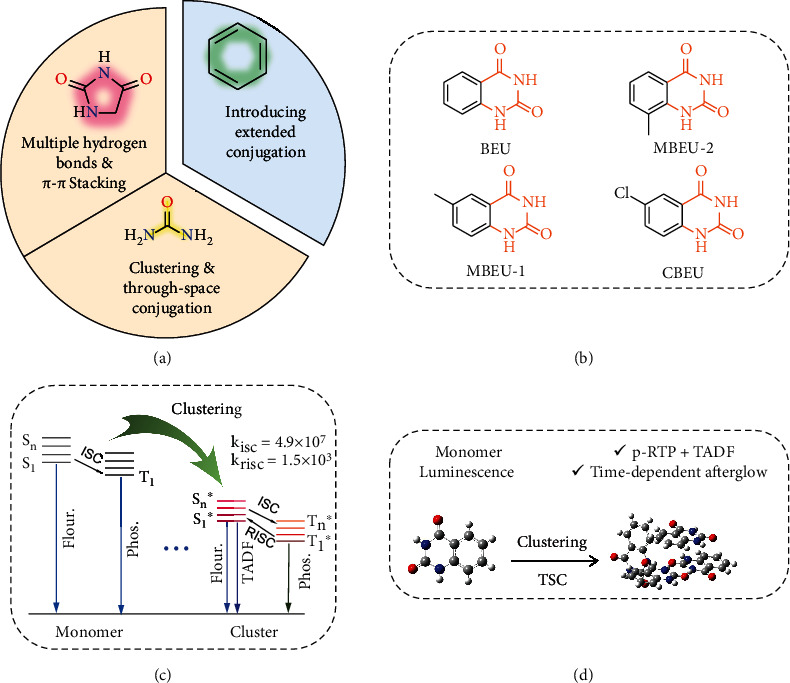
(a) Schematic demonstration of the design strategy by combining nonconventional luminophores and aromatics. (b) Chemical structures of the compounds studied herein. (c) Jablonski diagram and (d) schematic illustration of the monomer and cluster emissions.

In this work, by introducing nonconventional luminophores into aromatic structures (Figure 1(a)), we reported a luminophore, namely, benzoyleneurea (BEU), with distinct TADF and p-RTP emissions, illustrating time-dependent afterglow. For comparison, its derivatives of 6-methylquinazoline-2,4(1H,3H)-dione (MBEU-1), 8-methylquinazoline-2,4(1H,3H)-dione (MBEU-2), and 6-chloroquinazoline-2,4(1H,3H)-dione (CBEU) were also studied (Figure 1(b)). The introduction of ureide moiety into aromatic systems could not only promote SOC but further help generates effective intermolecular interactions to rigidify the conformation, thus favoring triplet emission. Moreover, it could also promote the emission of molecular aggregates and produce multiple emissive species within single component crystals. By adjusting the lifetime and *λ*_em_ of multiple emission species, it is then possible to access time-dependent afterglow. The CTE mechanism is adopted to explain the concurrence of diverse emission processes (Figures 1(c) and 1(d)). Furthermore, such a novel time-dependent afterglow luminophore can be well applied in advanced anticounterfeiting and encryption.

## 2. Results

**Figure 2:**
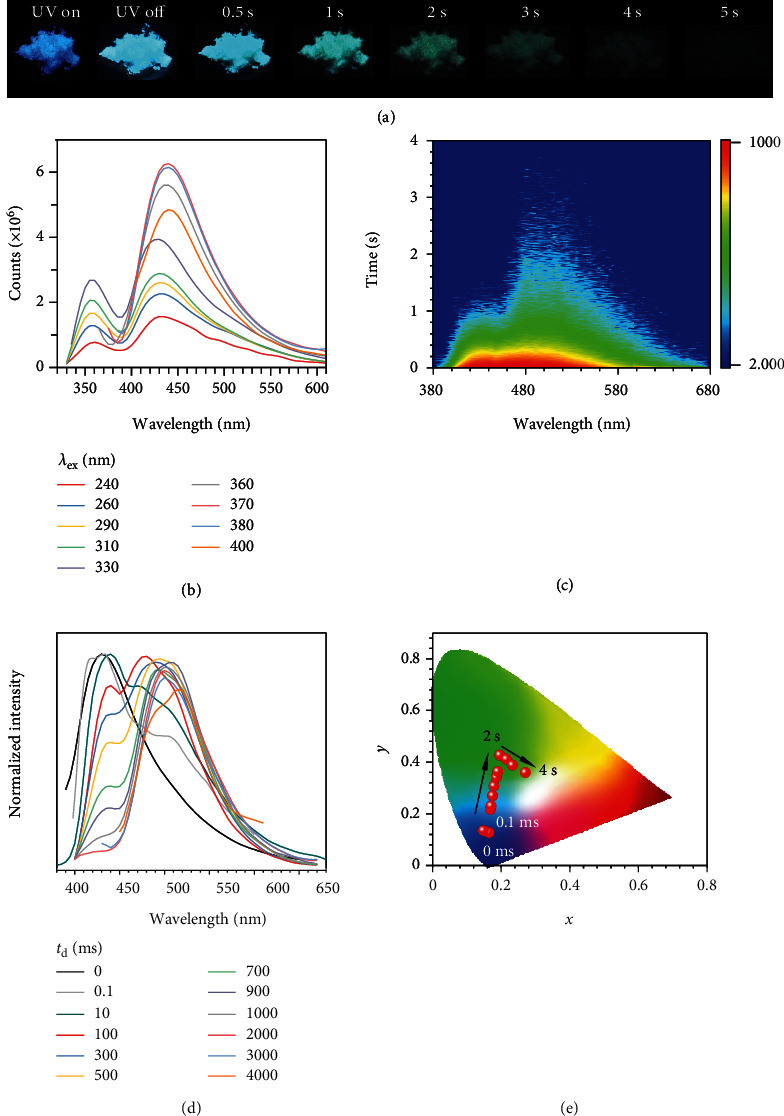
Photophysical properties of BEU crystals. (a) Photographs of BEU single-crystals taken with 312 UV light or after ceasing the irradiation at different time points. (b) Prompt emission spectra (*t*_d_ = 0 ms) of BEU crystals with varying *λ*_ex_ values. (c) Time-dependent afterglow mapping of BEU crystals at room temperature (*λ*_ex_ = 340 nm). (d) Prompt emission and delayed emission spectra with different delayed time (*λ*_ex_ = 340 nm). (e) Graphic illustration of the different recorded afterglow colors in the CIE diagram.

All four compounds are commercially available and utilized after careful recrystallization to exclude potential impurities. The chemical structure was verified by the ^1^H and ^13^C nuclear magnetic resonance (NMR) spectroscopy (Figure S1, S2), high-performance liquid chromatography (HPLC) (Figure S3 and S4), and single-crystal crystallography (Table S1). Unless specified, all measurements and observations were conducted on single crystals. As shown in Figure 2(a), at room temperature, BEU crystals emit bright blue light under a 312 nm UV lamp, and a long afterglow of up to 5 s can be observed when the UV lamp is off. Intriguingly, it can be clarified from Video S1 that the afterglow colors of the BEU crystals gradually change from cyan to green, showing a time-dependent feature.

To trace the origin of the phenomenon, photophysical measurements of BEU single-crystals were performed at room temperature. As illustrated in Figure 2(b), within the excitation range from 240 to 400 nm, prompt emission of BEU crystals exhibits two maxima at around 356 and 428 nm, which is consistent with the observed blue color. With increasing *λ*_ex_, the 356 nm peak almost unchanged, while the 428 nm peak gradually redshifts to 440 nm, featuring excitation-dependent characteristic. Time-resolved measurements reveal the existence of emissive species with ns-scale lifetime at 356 nm (Figure S5), indicative of its fluorescence nature. Further 3D mapping (Figure 2(c)) shows two emission bands with different decay profiles. In detail, the first one (400-450 nm) partially overlaps the prompt fluorescence band, implying the possibility of fluorescence-phosphorescence dual emission at this band. The second one is much broader (450-600 nm) and shows a slower decay profile (Figures 2(c) and 2(d)). In detail, with a delay time (t_d_) of 0.1 ms, the main peaks at 417 and 434 nm as well as a shoulder at 500 nm are noticed (Figure 2(d)) and the short wavelength emission (417 and 434 nm) is the main component at the delayed spectra (*t*_d_ = 0.1 ms). However, with a t_d_ of 100 ms, the 417 nm peak disappears and only two peaks (434 and 500 nm) are observed. With an increase in t_d_, the portion of the 434 nm peak is gradually decreased, and that at 500 nm becomes predominant. 2 s later, the 434 nm peak could hardly be observed and only the wideband at around 500 nm is noticed, which is significantly red-shifted compared with the main peak of PL emission. An emerging peak at around 517 nm is found with further prolonged t_d_, thus confirming the diversified emissive species. The Commission Internationale de l'Eclairage (CIE) coordinates of the emission at different times prove the color changing from deep blue (UV on) to light blue (*t*_d_ = 100 ms) and then to green (*t*_d_ = 2 s), with coordinates of (0.16, 0.13), (0.17, 0.22), and (0.20, 0.43), respectively. Therefore, a change in the proportions of different emission bands results in the observed time-dependent color-tunable afterglow.

**Figure 3:**
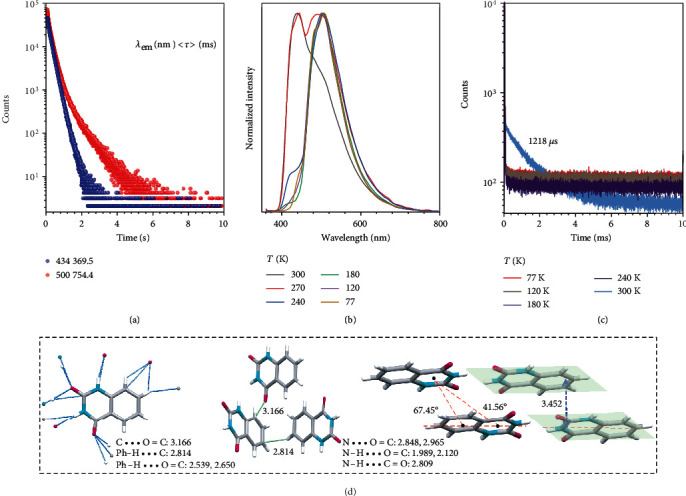
(a) p-RTP lifetimes of BEU crystals at different *λ*_em_*s* (*λ*_ex_ = 340 nm). (b) Delayed emission spectra (*t*_d_ = 0.1 ms) and (c) lifetime decay profiles monitored at 417 nm of BEU crystals at different temperatures (*λ*_ex_ = 340 nm). (d) Single-crystal structure and intermolecular interactions of BEU.

To further check the origin of those distinct emission bands, lifetime measurements were conducted at different time scales and varying temperatures. For those at 434 and 500 nm, lifetimes of up to 369.5 and 754.4 ms were recorded (Figure 3(a), Table S2), respectively, indicative of their p-RTP nature. Upon cooling to 77 K, refined peaks at 434, 484, 500, and 509 nm were observed under different *λ*_ex_*s*, with lifetimes prolonged to 712.3 (*λ*_em_ = 434 nm) and 1355.0 ms (*λ*_em_ = 500 nm) (Figure S6, Table S3), which further proves the phosphorescence nature of those emissions. Meanwhile, the 417 nm peak disappeared at 77 K while its intensity was gradually enhanced with an increase in temperature, demonstrating a typical TADF feature (Figure 3(b)). Moreover, the prompt fluorescence lifetime at 428 nm is 10.3 ns, which is much longer than that at 356 nm (1.26 ns), as shown in Figure S5. This indicates the peak at 428 nm may have a more complicated nature (with both prompt and delayed fluorescence) [24], compared with the emission at 356 nm (prompt fluorescence). Further lifetime measurements at different temperatures also prove the TADF nature. At 300 K, a lifetime of 1218 *μ*s is recorded, while no obvious decay is noticed at much lower temperatures (Figure 3(c)). The relatively short lifetime of TADF makes it impossible to contribute to the afterglow emissions; it thus can be concluded that the time-dependent afterglow originates from p-RTP emissions with comparable but different lifetimes (<*τ*>_*p*_). In contrast, at 77 K, due to the lack of TADF, the afterglow signal at 0.1 ms has a significant red-shift compared to that at room temperature (Figure S6). Meanwhile, since the emission at 500 nm becomes predominant, the cryogenic afterglow is mainly greenish, as shown in both delayed spectra and photographs (Figure S7).

To gain more insights into the provenance of multiple p-RTP and TADF emissions of BEU crystals, the PL properties of BEU solutions were scrutinized. When excited by 312 nm UV irradiation, the main emission peak of the dilute solution (10^−5^ M) is ~354 nm, which is ascribed to the monomer emission (Figure S8(a)). As the solution concentration increases, the spectra peak intensity at 354 nm reaches its maximum at 10^−4^ M and then notably decreases, indicative of an identical concentration quenching effect. In contrast, when the *λ*_ex_ is 350 nm, a new emission peak gradually appears at 417 nm with growing intensity (Figure S8(b)). This significant intensity enhancement can be clarified by the photographs taken under the irradiation of a 365 nm UV lamp (Figure S9), which should be caused by the molecular aggregation in concentrated solutions. Notably, for the concentrated solution (0.1 M), various maxima are recorded with changing *λ*_ex_*s*, indicative of typical excitation-dependent emission. In a dilute solution (e.g., 10^−5^ M), BEU molecules exist in isolated states, which exhibit monomer excitation and emission. In concentrated solutions (e.g., 0.1 M), owing to the aggregation of BEU molecules, diverse emissive clusters may form, whose emission peaks are bathochromically shifted to the visible region. Furthermore, excitation-dependent PL maxima are noticed due to the heterogeneous nature of the aggregates (Figure S10).

The previous results of concentration-enhanced emission phenomenon and excitation-dependent PL of BEU resemble those observed in nonconventional luminophores [36, 37, 42]. These phenomena could be understood on the basis of the CTE mechanism, specifically the clustering of BEU molecules (including ureide [35, 36] and benzene [44] moieties) could result in effective TSC among molecules, which yield diversified clustered luminophores with enriched and reduced energy levels together with narrowed energy gaps, thus facilitating the formation of multiple emissive centers. Meanwhile, adequate intermolecular interactions can stiffen the conformations of diversified clusters, which stabilizes triplet excitons and brings p-RTP emissions. Therefore, for the prompt emission of BEU crystals, the monomer fluorescence is peaking at 356 nm, while that around 417 nm should be attributed to both the prompt and delayed fluorescence of the clustered luminophores. For delayed emission, p-RTPs at 434, 500, and 517 nm should originate from different clustered luminophores, which is significantly redshifted compared with that of the monomer phosphorescence monitored from 10^−5^ M molecular solution at 77 K (*λ*_em_ = 400 nm, Figure S11).

To acquire a better understanding on the molecular packing, single-crystal structure and packing of BEU was determined. As shown in Figure 3(d), BEU molecules endorse a rigid planar conformation, with powerful N-H‧‧‧O=C (1.989, 2.120 Å) and C=O‧‧‧H-Ph (2.539, 2.650 Å) hydrogen bonds among ureides and benzene rings. Moreover, Ph-H‧‧‧C (2.539, 2.650 Å) and C‧‧‧O=C (3.166 Å) intermolecular interactions are also present, which could not only help rigidify molecular conformation, thus suppressing vibrational dissipations, but also bring about extended electron delocalization through effective TSC among different moieties. Specifically, the planar conformation of BEU also promotes interlayer *π* − *π* interactions (3.452 Å) [45] between the benzene ring and ureide group, which contribute to the extended electron delocalization and stabilization of the triplets.

**Figure 4:**
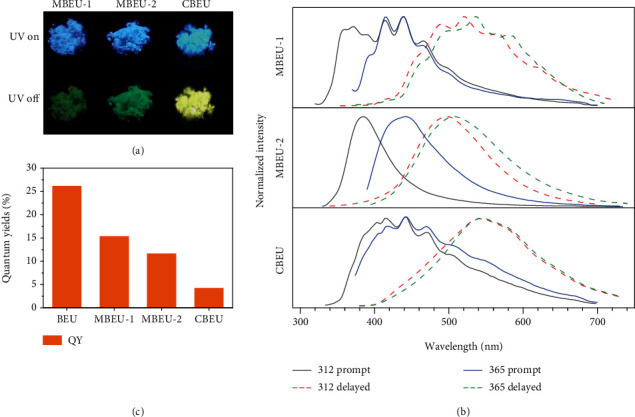
Photophysical properties of MBEU-1, MBEU-2, and CBEU crystals. (a) Photographs of three crystals taken under the irradiation of 312 UV light or after ceasing the UV light. (b) Prompt (*t*_d_ = 0 ms) and delayed emission (*t*_d_ = 1 ms) spectra of three crystals at different *λ*_ex_*s*. (c) *Φ*_*c*_ values of the four crystals (*λ*_ex_ = 365 nm).

The above multiemission of BEU single crystals inspired us to access an in-depth understanding of the origination of multiple p-RTP and time-dependent afterglow. We thus further investigated the photophysical properties of BEU derivatives of MBEU-1, MBEU-2, and CBEU. Under 312 nm UV irradiation, MBEU-1, MBEU-2, and CBEU crystals generate blue emissions, and moreover green, green, and yellow afterglows (Figure 4(a)) after the stop of irradiation, respectively. Their prompt emission demonstrates maxima at 357/373/391/413/439/468, 384, and 415/441/472 nm (Figure 4(b)), respectively. Meanwhile, with a t_d_ of 1 ms, they display PL maxima at 536/580, 510, and 539/594 nm, with lifetimes of 128.7/102.0, 357.2, and 62.9/53.1 ms (Figure S10), respectively, suggestive of their p-RTP features. These results, on one hand, verify the existence of diversified emissive species in crystals, moreover, illustrate the possibility of introducing nonconventional chromophores into aromatics to acquire multiple luminescent populations in aggregates. When excited with 365 nm UV light, for MBEU-1 and CBEU crystals, prompt PL profiles resembling to those excited by 312 nm are noticed, while a distinct spectrum with maxima at 421/445 nm is found for MBEU-2 crystals. Their delayed emissions, however, are slightly red-shifted in comparison to those with *λ*_ex_ of 312 nm (Figure 4(b)), on account of the various responses to excitation of varying emissive species. Notably, although these crystals show multiple p-RTP as well, the rather approaching lifetimes of different species make it difficult to observe time-dependent afterglow. In attempt to quantitatively analyze the emission of the organics, their quantum yields (*Φ*_*c*_) were demonstrated. The *Φ*_*c*_ values of MBEU-1 (15.3%), MBEU-2 (11.6%), and CBEU (4.2%) are much lower than that of BEU (26.1%) (Figure 4(c)), strongly indicative of the impact of molecular structure and molecular packing on photophysical properties. Moreover, peak resolving was conducted and phosphorescence quantum yields (*Φ*_*p*_) of these compounds were obtained. The *Φ*_*p*_ values also illustrate that BEU has the highest *Φ*_*p*_ among the four compounds, with *Φ*_*p*_ of 11.3%, 3.7%, 5.2%, and 2.4% for BEU, MBEU-1, MBEU-2, and CBEU, respectively.

**Figure 5:**
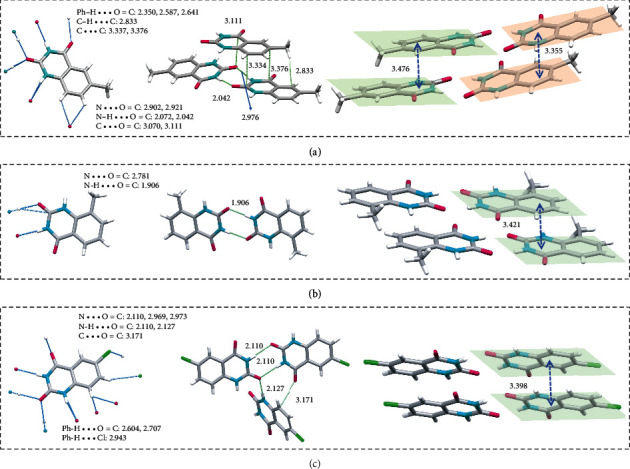
Single-crystal structures and fragmental molecular packing with denoted intermolecular interactions of (a) MBEU-1, (b) MBEU-2, and (c) CBEU.

Single-crystal structures of the three derivatives were studied, to obtain further insights into the structure-property relationship. As depicted in Figure 5, similar to BEU, multiple hydrogen bonds are formed in crystals, due to the occurrence of ureide moieties. The planer conformation of the three derivatives also promotes interlayer *π* − *π* interactions of 3.476/3.355, 3.421, and 3.398 Å, respectively. However, for MBEU-1, a 13.4° twist emerges between neighboring molecules in the same layer (Figure S13), which affects the intensity of hydrogen bonding (2.042, 2.120 Å) as well as weakens the tight interlayer molecular packing. For MBEU-2, the steric hindrance of the methyl group blocks the tight N-H‧‧‧H-PH hydrogen bonds between the carbonyl group and the benzene ring, which weakens the electron delocalization. In general, the crystal density of MBEU-1 and MBEU-2 (1.457, 1.441 g/cm3) is lower than that of BEU (1.507 g/cm^3^), indicative of the looser arrangement, resulting in the slightly shorter <*τ*>_*p*_*s* and decreased *Φ*_*c*_*s*. Specifically, for CBEU, the heavy atom effect and adequate lone pair (*n*) electrons of chlorine will invoke SOC and consequent intersystem crossing process, contributing to the plentiful triplet excitons. Accordingly, the RTP emission red-shifts to the yellowish zone and the UV absorption significantly widens compared to the other three substances (Figure S14), while the <*τ*>_*p*_ and *Φ*_*c*_ decline drastically due to the vulnerability of triplets. These results not only illustrate the influence of molecular structure on the single-crystal arrangement and aggregate emission but also show the potential tunability of this system by molecular design.

**Figure 6:**
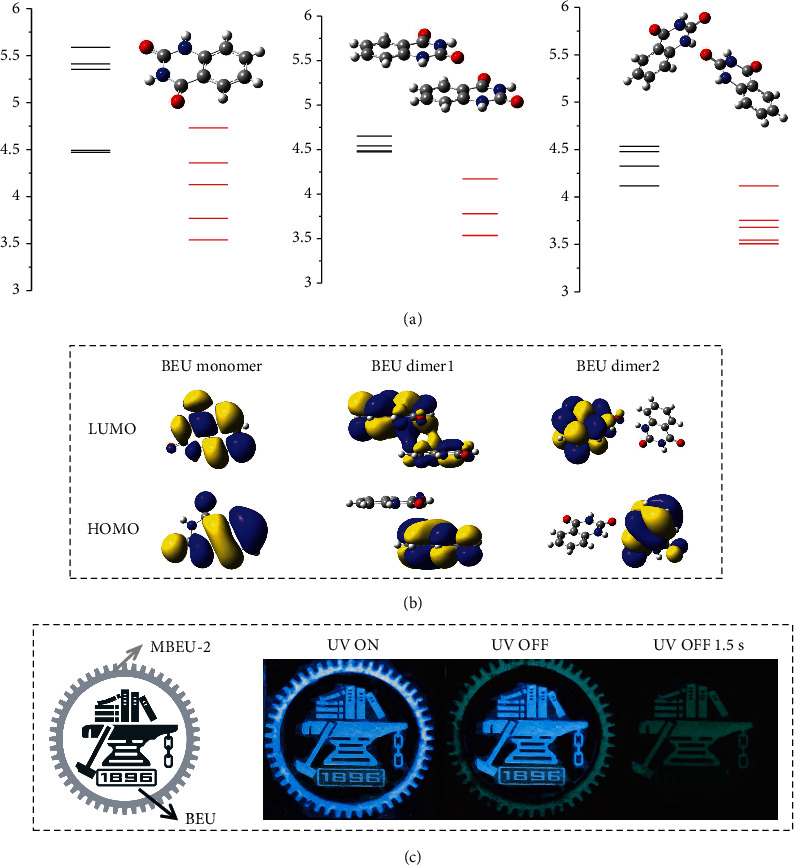
(a) Excited state energy levels of monomer and dimers of BEU. (b) The electron density distributions of HOMO and LUMO of monomer and dimers of BEU. (c) Demonstration of advanced anticounterfeiting and data encryption utilizing BEU and MBEU-2 crystals (*λ*_ex_ = 365 nm).

Time-dependent density functional theory (TD-DFT) calculation was conducted to gain further insights. As demonstrated in Figure 6(a), the excited state energy levels of the dimers are lower than those of the monomer, suggestive of the positive effect of aggregation on the red-shifted emissions. Moreover, when selecting dimer models with distinct intermolecular interactions, their energy levels are different. The divergent interactions between molecules may have different effects on the PL of molecular aggregates. Therefore, with the synergistic interplay of different molecular packing and diversified intermolecular interactions among the *π* and *n* electrons, clusters the energy level of molecular can be significantly reduced, and even multiple emission centers could appear. The calculated HOMO and LUMO electron densities (Figure 6(b)) also demonstrate the various electronic communication in different aggregates. In detail, remarkable TSC emerges between the dimer with sufficient *π* − *π* interaction, which could help reduce the energy level as well as rigidify the molecular conformation. The dimer constructed by hydrogen bonding between the phenyl and carbonyl depicts significant intermolecular charge transfer. Similar calculation results are also obtained for the other three derivatives, proving that the combination of nonconventional chromophores and the benzene ring promotes the intermolecular electron delocalization and thus reduces the energy levels of excitons (Figure S15-S17). In detail, for all three compounds, sufficient electron cloud overlap emerges in the LUMOs of dimers, indicative of the TSC nature at the excited states. Moreover, the energy levels of the aggregates are significantly lower than those of the monomers, which illustrates the formation of aggregates could help reduce the excited energy levels.

Benefiting from the bright PL, p-RTP, and moreover time-dependent afterglow, BEU crystals hold great potential for advanced anticounterfeiting and data encryption. As demonstrated in Figure 6(c), a pattern of SJTU Logo with a gear outside and a badge in the center is composed of MBEU-2 and BEU crystalline powders, respectively. Upon 365 nm UV irradiation, the whole pattern demonstrates analogous bluish PL. Upon turning off the UV excitation, the pattern instantly changes to a two-color mode with green gears and a blue badge. 1.5 s later, on account of the shorter <*τ*>_*p*_ of MBEU-2 crystals, the green afterglow gradually faded, while the badge part turned green due to the time-dependent afterglow of BEU crystals. Consequently, only the green badge inside is visible. Such time-dependent afterglow significantly improves confidentiality of the encryption and anticounterfeiting, compared with traditional single-color p-RTP materials.

## 3. Discussion

In conclusion, by introducing nonconventional luminophores into aromatic structures, a purely organic luminophore, namely, BEU, with time-dependent persistent afterglow in crystals, was reported in this work. It is figured out that BEU crystals possess multiple singlet and triplet emissions, including TADF and diversified p-RTP. Distinct lifetimes and variant relative intensity of p-RTP render BEU crystals with distinct time-dependent afterglow colors. Moreover, its methyl- or chloro-substituted derivatives also demonstrate multiple emission centers. This phenomenon could be reasonably explained in view of the CTE mechanism, namely, the clusterization of the molecules brings about diversified emissive species with varying TSCs, which can afford bright fluorescence and phosphorescence thanks to their sufficiently rigidified conformations [46–50]. Single-crystal data and calculation results show that the introduction of nonconventional moieties could construct multiple intermolecular interactions among electron-rich moieties, thus facilitating extended electron delocalization. Consequently, diverse clustered chromophores are formed, which lead to the multiple emission. The time-dependent afterglow color variation of BEU crystals grants its potential applications in advanced anticounterfeiting and encryption fields. These results also implicate the possibility to create new multifunctional luminophores through the combination of nonconventional and classic luminescent building blocks, which would inspirit further rational molecular design of new luminophores for emerging applications.

## Data Availability

The full version of data used to support the findings of this work is included online within the article and the supplementary materials.

## Supplementary Materials

Supplementary Materials Supplementary 1. Figure S1: ^1^H NMR spectrum of (a) BEU, (b) MBEU-1, (c) MBEU-2, and (d) CBEU in DMSO-d_6_. Figure S2: ^13^C NMR spectrum of (a) BEU, (b) MBEU-1, (c) MBEU-2, and (d) CBEU in DMSO-d_6_. Figure S3: HPLC diagrams of the four compounds. Figure S4: photographs of BEU solid purified by HPLC under 312 nm UV excitation or after ceasing the irradiation. Table S1: single crystal data of BEU, MBEU-1, MBEU-2, and CBEU. Figure S5: Ns-lifetime decay of BEU crystals at (a) 365 nm and (b) 428 nm. Table S2: RTP lifetimes and their value proportions of BEU crystals. Figure S6: (a) delayed emission spectra (*t*_d_ = 1 ms) of BEU crystals at 77 K with different *λ*_ex_*s*. (b) Lifetime decays of BEU crystals at different *λ*_em_*s* at 77 K (*λ*_ex_ = 340 nm). Table S3: cryogenic phosphorescence lifetimes and their value proportions of BEU crystals. Figure S7: (a) delayed emission spectra of BEU with different delayed times (*λ*_ex_ = 340 nm) at 77 K. (b) CIE coordinates of the recorded spectra. Figure S8: emission spectra of gradient BEU/DMF solutions with (a) *λ*_ex_ = 312 nm and (b) *λ*_ex_ = 350 nm and their corresponding excitation spectra with (a) *λ*_em_ = 354 nm and (b) *λ*_em_ = 417 nm. Figure S9: photographs of gradient BEU/DMF solutions under (a) 312 nm UV and (b) 365 nm UV with *Φ*_*c*_ values (*λ*_ex_ = 365 nm). Figure S10: emission spectra of 0.1 M BEU/DMF solutions with different *λ*_ex_*s*. Figure S11: prompt and delayed emission spectra of 10^−5^ M BEU/DMF solution at 77 K. (*λ*_ex_ = 312 nm, *t*_d_ = 0.1 ms) Figure S12: lifetimes of p-RTP for (a) MBEU-1, (b) MBEU-2, and (c) CBEU crystals at different *λ*_em_*s* (*λ*_ex_ = 312 nm). Figure S13: schematic illustration of the twisted molecular conformation of MBEU-1 crystals in the same layer. Figure S14: UV absorption of BEU, MBEU-1, MBEU-2, and CBEU crystals. Figure S15: (a) electron density distributions of HOMO and LUMO of MBEU-1 monomer and dimers. (b) Energy levels of MBEU-1 monomer and dimers. Figure S16: (a) electron density distributions of HOMO and LUMO of MBEU-2 monomer and dimers. (b) Energy levels of MBEU-2 monomer and dimers. Figure S17: (a) electron density distributions of HOMO and LUMO of CBEU monomer and dimers. (b) Energy levels of CBEU monomer and dimers. Supplementary 2. Video S1: the recorded time-dependent afterglow of BEU crystals. Supplementary 3: BEU.cif single-crystal structure of BEU. Supplementary 4: MBEU-1.cif single-crystal structure of MBEU-1. Supplementary 5: MBEU-2.cif single-crystal structure of MBEU-2. Supplementary 6: CBEU.cif single-crystal structure of CBEU. Supplementary 7: Checkcif document of the crystals.
