# Supplementary material for: Time-Dependent Afterglow from a Single Component Organic Luminogen
Source: Research (Wash D C). 2021 Aug 27;2021:9757460. doi: 10.34133/2021/9757460 (PMC8422276; doi:10.34133/2021/9757460)

BEU

checkCIF/PLATON report

Structure factors have been supplied for datablock(s) t_a

THIS REPORT IS FOR GUIDANCE ONLY. IF USED AS PART OF A REVIEW PROCEDURE FOR PUBLICATION, IT SHOULD NOT REPLACE THE EXPERTISE OF AN EXPERIENCED CRYSTALLOGRAPHIC REFEREE.

No syntax errors found. CIF dictionary Interpreting this report

Datablock: t_a

```
Bond precision:  C-C = 0.0018 A          Wavelength=1.54178

Cell:            a=10.8541(5)     b=5.2696(3)        c=12.6736(6)
                 alpha=90         beta=99.598(2)     gamma=90
Temperature:     294 K

                Calculated                 Reported
Volume          714.74(6)                  714.74(6)
Space group     P 21/c                     P 21/c
Hall group      -P 2ybc                    -P 2ybc
Moiety formula  C8 H6 N2 O2                C8 H6 N2 O2
Sum formula     C8 H6 N2 O2                C8 H6 N2 O2
Mr              162.15                     162.15
Dx,g cm-3       1.507                      1.507
Z               4                          4
Mu (mm-1)       0.939                      0.939
F000            336.0                      336.0
F000'           337.15
h,k,lmax        13,6,15                    13,6,15
Nref            1316                       1316
Tmin,Tmax       0.829,0.829                0.659,0.753
Tmin'           0.829

Correction method= # Reported T Limits: Tmin=0.659 Tmax=0.753
AbsCorr = ?

Data completeness= 1.000          Theta(max)= 68.244

R(reflections)= 0.0342( 1134)     wR2(reflections)= 0.1031( 1316)

S = 1.035                   Npar= 109
```

The following ALERTS were generated. Each ALERT has the format
test-name_ALERT_alert-type_alert-level.
Click on the hyperlinks for more details of the test.

Alert level C

```
PLAT052_ALERT_1_C Info on Absorption Correction Method   Not Given     Please Do !
PLAT978_ALERT_2_C Number C-C Bonds with Positive Residual Density.           0 Info
```

Alert level G

```
PLAT007_ALERT_5_G Number of Unrefined Donor-H Atoms .............           2 Report
PLAT883_ALERT_1_G No Info for _atom_sites_solution_primary .......     Please Do !
```

```
   0 ALERT level A = Most likely a serious problem - resolve or explain
   0 ALERT level B = A potentially serious problem, consider carefully
   2 ALERT level C = Check. Ensure it is not caused by an omission or oversight
   2 ALERT level G = General information/check it is not something unexpected

   2 ALERT type 1 CIF construction/syntax error, inconsistent or missing data
   1 ALERT type 2 Indicator that the structure model may be wrong or deficient
   0 ALERT type 3 Indicator that the structure quality may be low
   0 ALERT type 4 Improvement, methodology, query or suggestion
   1 ALERT type 5 Informative message, check
```

It is advisable to attempt to resolve as many as possible of the alerts in all categories. Often the minor alerts point to easily fixed oversights, errors and omissions in your CIF or refinement strategy, so attention to these fine details can be worthwhile. In order to resolve some of the more serious problems it may be necessary to carry out additional measurements or structure refinements. However, the purpose of your study may justify the reported deviations and the more serious of these should normally be commented upon in the discussion or experimental section of a paper or in the "special_details" fields of the CIF. checkCIF was carefully designed to identify outliers and unusual parameters, but every test has its limitations and alerts that are not important in a particular case may appear. Conversely, the absence of alerts does not guarantee there are no aspects of the results needing attention. It is up to the individual to critically assess their own results and, if necessary, seek expert advice.

Publication of your CIF in IUCr journals

A basic structural check has been run on your CIF. These basic checks will be run on all CIFs submitted for publication in IUCr journals (*Acta Crystallographica*, *Journal of Applied Crystallography*, *Journal of Synchrotron Radiation*); however, if you intend to submit to *Acta Crystallographica Section C* or *E* or *IUCrData*, you should make sure that full publication checks are run on the final version of your CIF prior to submission.

Publication of your CIF in other journals

Please refer to the *Notes for Authors* of the relevant journal for any special instructions relating to CIF submission.

PLATON version of 18/02/2019; check.def file version of 18/02/2019

Datablock t_a - ellipsoid plot

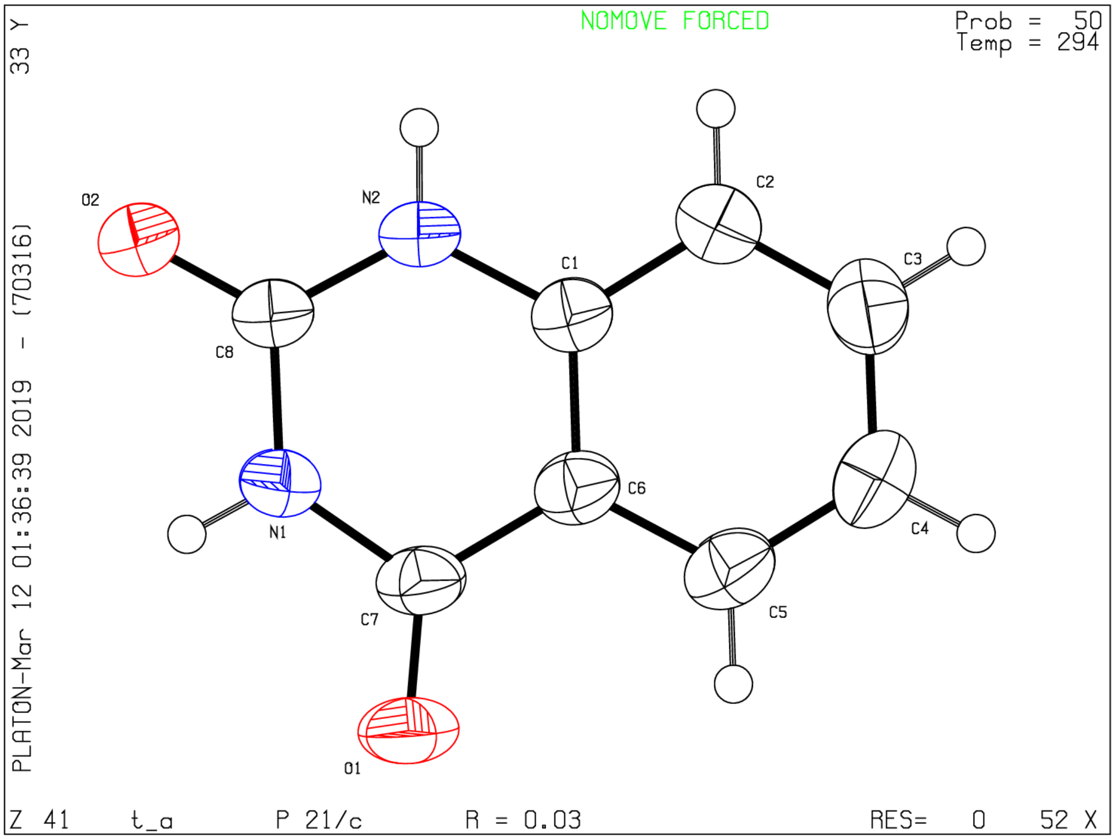

checkCIF/PLATON report

Structure factors have been supplied for datablock(s) ta_a

THIS REPORT IS FOR GUIDANCE ONLY. IF USED AS PART OF A REVIEW PROCEDURE FOR PUBLICATION, IT SHOULD NOT REPLACE THE EXPERTISE OF AN EXPERIENCED CRYSTALLOGRAPHIC REFEREE.

No syntax errors found. CIF dictionary Interpreting this report

Datablock: ta_a

```
Bond precision:  C-C = 0.0027 A          Wavelength=1.54178

Cell:            a=14.0230(16)     b=3.8795(4)      c=14.0230(16)
                 alpha=90          beta=94.2600     gamma=90
Temperature:     293 K

                Calculated                 Reported
Volume          760.78(15)                 760.77(15)
Space group     P 21/c                     P 21/c
Hall group      -P 2ybc                    -P 2ybc
Moiety formula  C8 H5 Cl N2 O2             C8 H5 Cl N2 O2
Sum formula     C8 H5 Cl N2 O2             C8 H5 Cl N2 O2
Mr              196.59                     196.59
Dx,g cm-3       1.716                      1.716
Z               4                          4
Mu (mm-1)       4.162                      4.162
F000            400.0                      400.0
F000'           402.62
h,k,lmax        16,4,16                    16,4,16
Nref            1267                       1241
Tmin,Tmax       0.460,0.473                0.614,0.753
Tmin'           0.348

Correction method= # Reported T Limits: Tmin=0.614 Tmax=0.753
AbsCorr = ?

Data completeness= 0.979          Theta(max)= 63.693

R(reflections)= 0.0344( 1127)     wR2(reflections)= 0.1202( 1241)

S = 0.996                   Npar= 118
```

The following ALERTS were generated. Each ALERT has the format
test-name_ALERT_alert-type_alert-level.
Click on the hyperlinks for more details of the test.

Alert level B

```
SYMMS02_ALERT_1_B  The unit-cell lengths a and c should not be equal for a
            monoclinic cell
  Cell      14.0230    3.8795   14.0230
  Angles    90.0000   94.2600   90.0000
```

Alert level C

```
THETM01_ALERT_3_C  The value of sine(theta_max)/wavelength is less than 0.590
            Calculated sin(theta_max)/wavelength =    0.5814
PLAT029_ALERT_3_C _diffrn_measured_fraction_theta_full value Low .      0.979 Why?
PLAT052_ALERT_1_C Info on Absorption Correction Method   Not Given     Please Do !
PLAT911_ALERT_3_C Missing FCF Refl Between Thmin & STh/L=    0.581         26 Report
```

Alert level G

```
PLAT007_ALERT_5_G Number of Unrefined Donor-H Atoms ..............          2 Report
PLAT145_ALERT_4_G s.u. on beta     Small or Missing ..............     0.0000 Degree
PLAT199_ALERT_1_G Reported _cell_measurement_temperature ..... (K)        293 Check
PLAT200_ALERT_1_G Reported   _diffrn_ambient_temperature ..... (K)        293 Check
PLAT883_ALERT_1_G No Info/Value for _atom_sites_solution_primary .     Please Do !
PLAT909_ALERT_3_G Percentage of I>2sig(I) Data at Theta(Max) Still        88% Note
PLAT913_ALERT_3_G Missing # of Very Strong Reflections in FCF ....          3 Note
PLAT978_ALERT_2_G Number C-C Bonds with Positive Residual Density.          5 Info
```

0 **ALERT level A** = Most likely a serious problem - resolve or explain
1 **ALERT level B** = A potentially serious problem, consider carefully
4 **ALERT level C** = Check. Ensure it is not caused by an omission or oversight
8 **ALERT level G** = General information/check it is not something unexpected

5 ALERT type 1 CIF construction/syntax error, inconsistent or missing data
1 ALERT type 2 Indicator that the structure model may be wrong or deficient
5 ALERT type 3 Indicator that the structure quality may be low
1 ALERT type 4 Improvement, methodology, query or suggestion
1 ALERT type 5 Informative message, check

It is advisable to attempt to resolve as many as possible of the alerts in all categories. Often the minor alerts point to easily fixed oversights, errors and omissions in your CIF or refinement strategy, so attention to these fine details can be worthwhile. In order to resolve some of the more serious problems it may be necessary to carry out additional measurements or structure refinements. However, the purpose of your study may justify the reported deviations and the more serious of these should normally be commented upon in the discussion or experimental section of a paper or in the "special_details" fields of the CIF. checkCIF was carefully designed to identify outliers and unusual parameters, but every test has its limitations and alerts that are not important in a particular case may appear. Conversely, the absence of alerts does not guarantee there are no aspects of the results needing attention. It is up to the individual to critically assess their own results and, if necessary, seek expert advice.

Publication of your CIF in IUCr journals

A basic structural check has been run on your CIF. These basic checks will be run on all CIFs submitted for publication in IUCr journals (*Acta Crystallographica*, *Journal of Applied Crystallography*, *Journal of Synchrotron Radiation*); however, if you intend to submit to *Acta Crystallographica Section C* or *E* or *IUCrData*, you should make sure that full publication checks are run on the final version of your CIF prior to submission.

Publication of your CIF in other journals

Please refer to the *Notes for Authors* of the relevant journal for any special instructions relating to CIF submission.

PLATON version of 03/05/2019; check.def file version of 29/04/2019

Datablock ta_a - ellipsoid plot

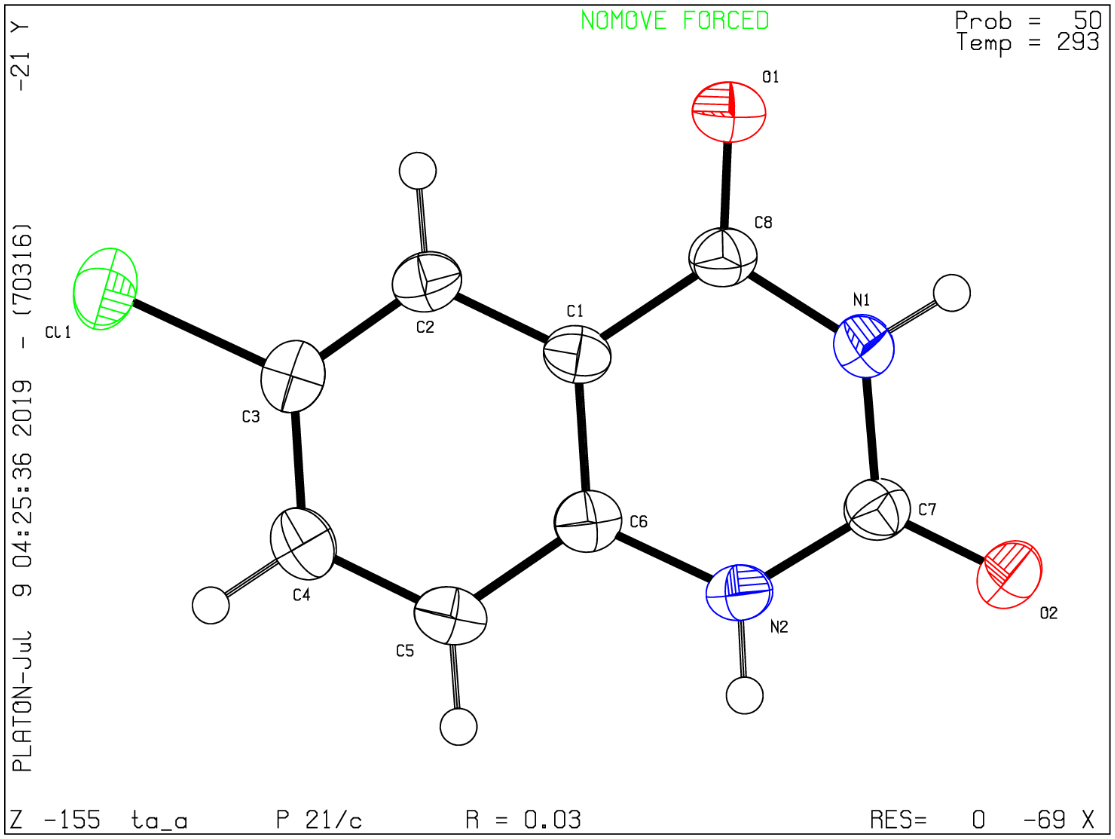

checkCIF/PLATON report

Structure factors have been supplied for datablock(s) ta_a

THIS REPORT IS FOR GUIDANCE ONLY. IF USED AS PART OF A REVIEW PROCEDURE FOR PUBLICATION, IT SHOULD NOT REPLACE THE EXPERTISE OF AN EXPERIENCED CRYSTALLOGRAPHIC REFEREE.

No syntax errors found.   CIF dictionary   Interpreting this report

Datablock: ta_a

```
Bond precision:  C-C = 0.0116 A          Wavelength=1.54178

Cell:            a=15.3058(12)     b=5.0105(4)      c=21.0179(16)
                 alpha=90          beta=94.7800     gamma=90
Temperature:     297 K

                Calculated                Reported
Volume          1606.3(2)                 1606.3(2)
Space group     P 21/n                    P 21/n
Hall group      -P 2yn                    -P 2yn
Moiety formula  C9 H8 N2 O2               C9 H8 N2 O2
Sum formula     C9 H8 N2 O2               C9 H8 N2 O2
Mr              176.17                    176.17
Dx,g cm-3       1.457                     1.457
Z               8                         8
Mu (mm-1)       0.881                     0.881
F000            736.0                     736.0
F000'           738.43
h,k,lmax        17,5,24                   17,5,24
Nref            2635                      2622
Tmin,Tmax       0.853,0.876               0.564,0.753
Tmin'           0.853

Correction method= # Reported T Limits: Tmin=0.564 Tmax=0.753
AbsCorr = ?

Data completeness= 0.995           Theta(max)= 63.689

R(reflections)= 0.1122( 1671)      wR2(reflections)= 0.3411( 2622)

S = 1.097                  Npar= 238
```

The following ALERTS were generated. Each ALERT has the format
test-name_ALERT_alert-type_alert-level.
Click on the hyperlinks for more details of the test.

Alert level B

```
PLAT340_ALERT_3_B Low Bond Precision on  C-C Bonds ...............    0.01162 Ang.
PLAT930_ALERT_2_B FCF-based   Twin Law ( 1 0 2)[ 1 0 1] Est.d BASF       0.18 Check
```

Alert level C

```
THETM01_ALERT_3_C  The value of sine(theta_max)/wavelength is less than 0.590
            Calculated sin(theta_max)/wavelength =    0.5814
PLAT052_ALERT_1_C Info on Absorption Correction Method   Not Given     Please Do !
PLAT082_ALERT_2_C High R1 Value ..................................       0.11 Report
PLAT084_ALERT_3_C High wR2 Value (i.e. > 0.25) ...................       0.34 Report
PLAT906_ALERT_3_C Large K Value in the Analysis of Variance ......     17.509 Check
PLAT906_ALERT_3_C Large K Value in the Analysis of Variance ......      4.641 Check
PLAT906_ALERT_3_C Large K Value in the Analysis of Variance ......      2.459 Check
PLAT911_ALERT_3_C Missing FCF Refl Between Thmin & STh/L=    0.581         13 Report
```

Alert level G

```
PLAT007_ALERT_5_G Number of Unrefined Donor-H Atoms ..............          4 Report
PLAT083_ALERT_2_G SHELXL Second Parameter in WGHT  Unusually Large      15.05 Why ?
PLAT145_ALERT_4_G s.u. on beta     Small or Missing ..............     0.0000 Degree
PLAT870_ALERT_4_G ALERTS Related to Twinning Effects Suppressed ..          ! Info
PLAT883_ALERT_1_G No Info/Value for _atom_sites_solution_primary .     Please Do !
PLAT931_ALERT_5_G CIFcalcFCF  Twin Law ( 1 0-1)         Est.d BASF       0.18 Check
PLAT933_ALERT_2_G Number of OMIT Records in Embedded .res File ...          4 Note
PLAT941_ALERT_3_G Average HKL Measurement Multiplicity ...........        4.8 Low
PLAT965_ALERT_2_G The SHELXL WEIGHT Optimisation has not Converged     Please Check
```

0 **ALERT level A** = Most likely a serious problem - resolve or explain
2 **ALERT level B** = A potentially serious problem, consider carefully
8 **ALERT level C** = Check. Ensure it is not caused by an omission or oversight
9 **ALERT level G** = General information/check it is not something unexpected

2 ALERT type 1 CIF construction/syntax error, inconsistent or missing data
5 ALERT type 2 Indicator that the structure model may be wrong or deficient
8 ALERT type 3 Indicator that the structure quality may be low
2 ALERT type 4 Improvement, methodology, query or suggestion
2 ALERT type 5 Informative message, check

It is advisable to attempt to resolve as many as possible of the alerts in all categories. Often the minor alerts point to easily fixed oversights, errors and omissions in your CIF or refinement strategy, so attention to these fine details can be worthwhile. In order to resolve some of the more serious problems it may be necessary to carry out additional measurements or structure refinements. However, the purpose of your study may justify the reported deviations and the more serious of these should normally be commented upon in the discussion or experimental section of a paper or in the "special_details" fields of the CIF. checkCIF was carefully designed to identify outliers and unusual parameters, but every test has its limitations and alerts that are not important in a particular case may appear. Conversely, the absence of alerts does not guarantee there are no aspects of the results needing attention. It is up to the individual to critically assess their own results and, if necessary, seek expert advice.

Publication of your CIF in IUCr journals

A basic structural check has been run on your CIF. These basic checks will be run on all CIFs submitted for publication in IUCr journals (*Acta Crystallographica*, *Journal of Applied Crystallography*, *Journal of Synchrotron Radiation*); however, if you intend to submit to *Acta Crystallographica Section C* or *E* or *IUCrData*, you should make sure that full publication checks are run on the final version of your CIF prior to submission.

Publication of your CIF in other journals

Please refer to the *Notes for Authors* of the relevant journal for any special instructions relating to CIF submission.

PLATON version of 22/03/2021; check.def file version of 19/03/2021

Datablock ta_a - ellipsoid plot

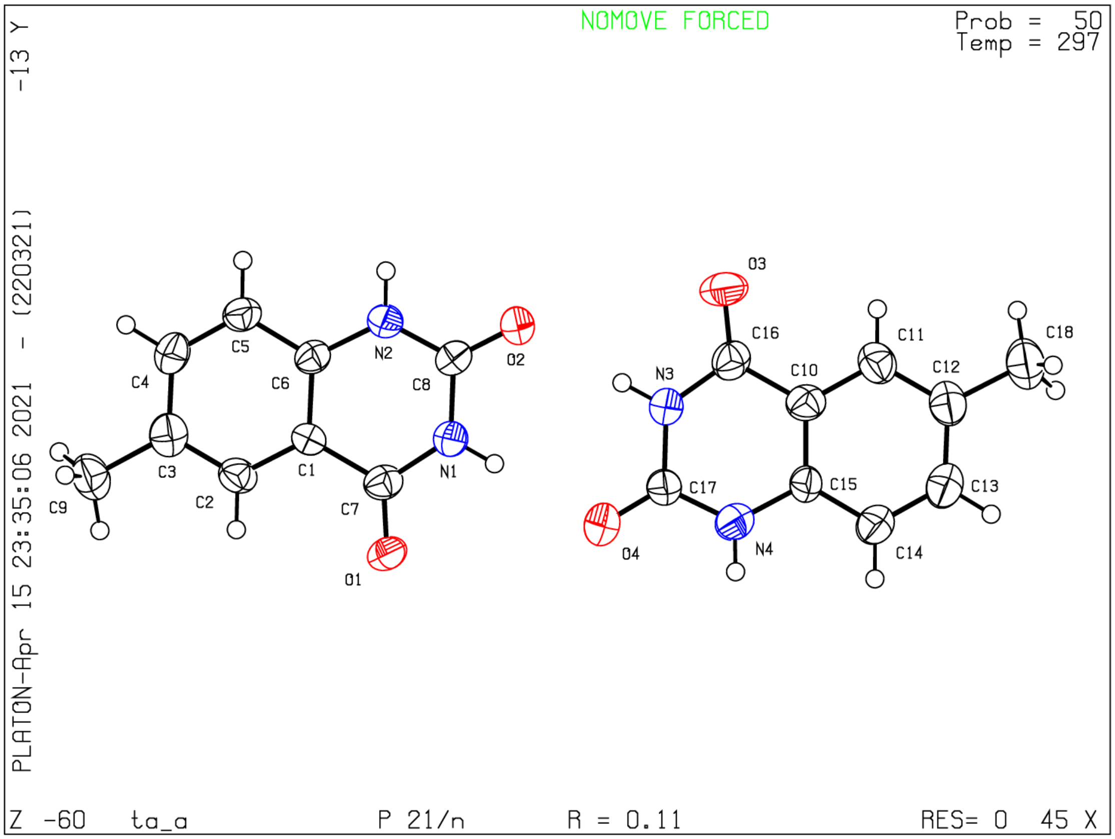

checkCIF/PLATON report

Structure factors have been supplied for datablock(s) ta_a

THIS REPORT IS FOR GUIDANCE ONLY. IF USED AS PART OF A REVIEW PROCEDURE FOR PUBLICATION, IT SHOULD NOT REPLACE THE EXPERTISE OF AN EXPERIENCED CRYSTALLOGRAPHIC REFEREE.

No syntax errors found.   CIF dictionary   Interpreting this report

Datablock: ta_a

```
Bond precision:  C-C = 0.0020 A          Wavelength=1.54178

Cell:            a=11.7154(6)     b=4.8996(3)       c=16.6621(9)
                 alpha=90         beta=110.5800     gamma=90
Temperature:     173 K

                Calculated                 Reported
Volume          895.38(9)                  895.36(9)
Space group     P 21/c                     P 21/c
Hall group      -P 2ybc                    -P 2ybc
Moiety formula  C9 H8 N2 O2, H2 O          C9 H8 N2 O2, H2 O
Sum formula     C9 H10 N2 O3               C9 H10 N2 O3
Mr              194.19                     194.19
Dx,g cm-3       1.441                      1.441
Z               4                          4
Mu (mm-1)       0.926                      0.926
F000            408.0                      408.0
F000'           409.41
h,k,lmax        14,5,20                    14,5,20
Nref            1644                       1633
Tmin,Tmax       0.831,0.878                0.596,0.753
Tmin'           0.831

Correction method= # Reported T Limits: Tmin=0.596 Tmax=0.753
AbsCorr = ?

Data completeness= 0.993          Theta(max)= 68.708

R(reflections)= 0.0363( 1367)     wR2(reflections)= 0.1029( 1633)

S = 1.025                  Npar= 140
```

The following ALERTS were generated. Each ALERT has the format
test-name_ALERT_alert-type_alert-level.
Click on the hyperlinks for more details of the test.

Alert level C

```
PLAT052_ALERT_1_C Info on Absorption Correction Method   Not Given     Please Do !
PLAT911_ALERT_3_C Missing FCF Refl Between Thmin & STh/L=    0.600          5 Report
```

Alert level G

```
PLAT007_ALERT_5_G Number of Unrefined Donor-H Atoms .............          1 Report
PLAT145_ALERT_4_G s.u. on beta     Small or Missing .............     0.0000 Degree
PLAT883_ALERT_1_G No Info/Value for _atom_sites_solution_primary .     Please Do !
PLAT912_ALERT_4_G Missing # of FCF Reflections Above STh/L=  0.600          6 Note
PLAT913_ALERT_3_G Missing # of Very Strong Reflections in FCF ....          3 Note
PLAT965_ALERT_2_G The SHELXL WEIGHT Optimisation has not Converged     Please Check
PLAT978_ALERT_2_G Number C-C Bonds with Positive Residual Density.          7 Info
```

```
   0 ALERT level A = Most likely a serious problem - resolve or explain
   0 ALERT level B = A potentially serious problem, consider carefully
   2 ALERT level C = Check. Ensure it is not caused by an omission or oversight
   7 ALERT level G = General information/check it is not something unexpected

   2 ALERT type 1 CIF construction/syntax error, inconsistent or missing data
   2 ALERT type 2 Indicator that the structure model may be wrong or deficient
   2 ALERT type 3 Indicator that the structure quality may be low
   2 ALERT type 4 Improvement, methodology, query or suggestion
   1 ALERT type 5 Informative message, check
```

It is advisable to attempt to resolve as many as possible of the alerts in all categories. Often the minor alerts point to easily fixed oversights, errors and omissions in your CIF or refinement strategy, so attention to these fine details can be worthwhile. In order to resolve some of the more serious problems it may be necessary to carry out additional measurements or structure refinements. However, the purpose of your study may justify the reported deviations and the more serious of these should normally be commented upon in the discussion or experimental section of a paper or in the "special_details" fields of the CIF. checkCIF was carefully designed to identify outliers and unusual parameters, but every test has its limitations and alerts that are not important in a particular case may appear. Conversely, the absence of alerts does not guarantee there are no aspects of the results needing attention. It is up to the individual to critically assess their own results and, if necessary, seek expert advice.

Publication of your CIF in IUCr journals

A basic structural check has been run on your CIF. These basic checks will be run on all CIFs submitted for publication in IUCr journals (*Acta Crystallographica*, *Journal of Applied Crystallography*, *Journal of Synchrotron Radiation*); however, if you intend to submit to *Acta Crystallographica Section C* or *E* or *IUCrData*, you should make sure that full publication checks are run on the final version of your CIF prior to submission.

Publication of your CIF in other journals

Please refer to the *Notes for Authors* of the relevant journal for any special instructions relating to CIF submission.

PLATON version of 05/12/2020; check.def file version of 05/12/2020

Datablock ta_a - ellipsoid plot

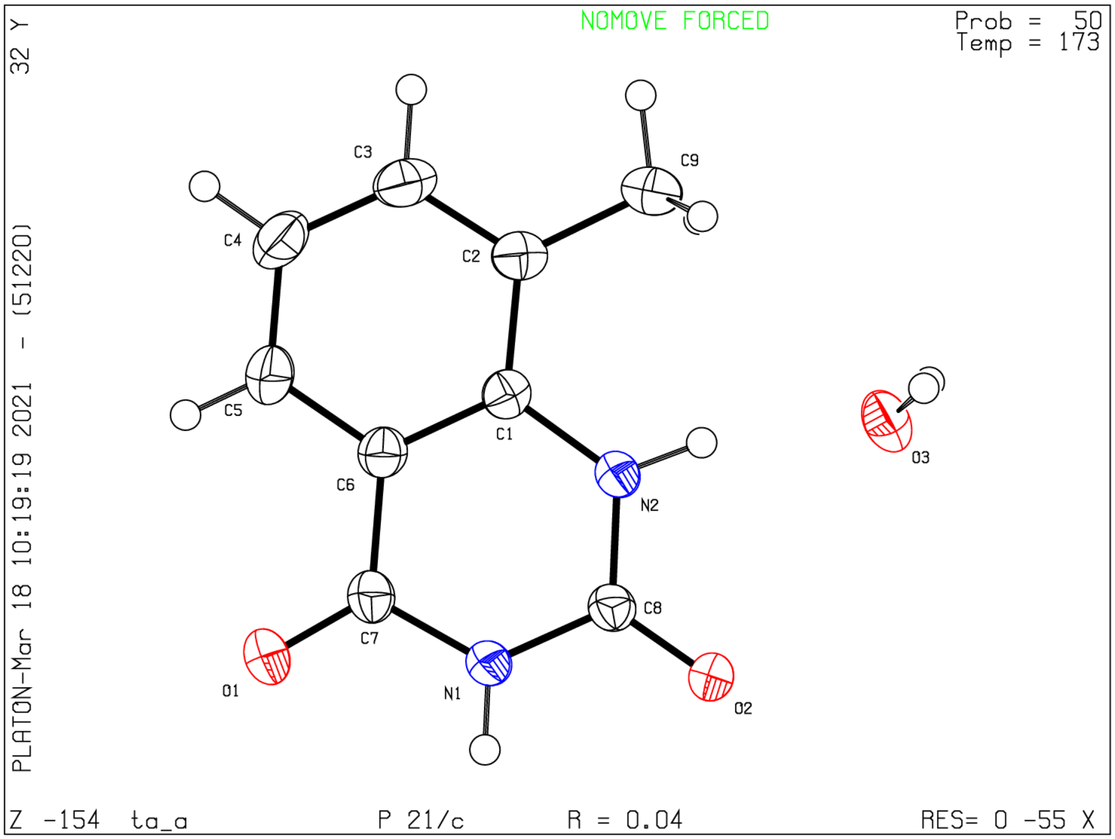

Supplement: Supplementary Materials — Supplementary 1. Figure S1: 1H NMR spectrum of (a) BEU, (b) MBEU-1, (c) MBEU-2, and (d) CBEU in DMSO-d6. Figure S2: 13C NMR spectrum of (a) BEU, (b) MBEU-1, (c) MBEU-2, and (d) CBEU in DMSO-d6. Figure S3: HPLC diagrams of the four compounds. Figure S4: photographs of BEU solid purified by HPLC under 312 nm UV excitation or after ceasing the irradiation. Table S1: single crystal data of BEU, MBEU-1, MBEU-2, and CBEU. Figure S5: Ns-lifetime decay of BEU crystals at (a) 365 nm and (b) 428 nm. Table S2: RTP lifetimes and their value proportions of BEU crystals. Figure S6: (a) delayed emission spectra (td = 1 ms) of BEU crystals at 77 K with different λexs. (b) Lifetime decays of BEU crystals at different λems at 77 K (λex = 340 nm). Table S3: cryogenic phosphorescence lifetimes and their value proportions of BEU crystals. Figure S7: (a) delayed emission spectra of BEU with different delayed times (λex = 340 nm) at 77 K. (b) CIE coordinates of the recorded spectra. Figure S8: emission spectra of gradient BEU/DMF solutions with (a) λex = 312 nm and (b) λex = 350 nm and their corresponding excitation spectra with (a) λem = 354 nm and (b) λem = 417 nm. Figure S9: photographs of gradient BEU/DMF solutions under (a) 312 nm UV and (b) 365 nm UV with Φc values (λex = 365 nm). Figure S10: emission spectra of 0.1 M BEU/DMF solutions with different λexs. Figure S11: prompt and delayed emission spectra of 10−5 M BEU/DMF solution at 77 K. (λex = 312 nm, td = 0.1 ms) Figure S12: lifetimes of p-RTP for (a) MBEU-1, (b) MBEU-2, and (c) CBEU crystals at different λems (λex = 312 nm). Figure S13: schematic illustration of the twisted molecular conformation of MBEU-1 crystals in the same layer. Figure S14: UV absorption of BEU, MBEU-1, MBEU-2, and CBEU crystals. Figure S15: (a) electron density distributions of HOMO and LUMO of MBEU-1 monomer and dimers. (b) Energy levels of MBEU-1 monomer and dimers. Figure S16: (a) electron density distributions of HOMO and LUMO of MBEU-2 monomer and dim [file 9757460.f1.zip › checkcif.pdf]
