# Supplementary material for: Time-Dependent Afterglow from a Single Component Organic Luminogen
Source: Research (Wash D C). 2021 Aug 27;2021:9757460. doi: 10.34133/2021/9757460 (PMC8422276; doi:10.34133/2021/9757460)
Supplement: Supplementary Materials — Supplementary 1. Figure S1: 1H NMR spectrum of (a) BEU, (b) MBEU-1, (c) MBEU-2, and (d) CBEU in DMSO-d6. Figure S2: 13C NMR spectrum of (a) BEU, (b) MBEU-1, (c) MBEU-2, and (d) CBEU in DMSO-d6. Figure S3: HPLC diagrams of the four compounds. Figure S4: photographs of BEU solid purified by HPLC under 312 nm UV excitation or after ceasing the irradiation. Table S1: single crystal data of BEU, MBEU-1, MBEU-2, and CBEU. Figure S5: Ns-lifetime decay of BEU crystals at (a) 365 nm and (b) 428 nm. Table S2: RTP lifetimes and their value proportions of BEU crystals. Figure S6: (a) delayed emission spectra (td = 1 ms) of BEU crystals at 77 K with different λexs. (b) Lifetime decays of BEU crystals at different λems at 77 K (λex = 340 nm). Table S3: cryogenic phosphorescence lifetimes and their value proportions of BEU crystals. Figure S7: (a) delayed emission spectra of BEU with different delayed times (λex = 340 nm) at 77 K. (b) CIE coordinates of the recorded spectra. Figure S8: emission spectra of gradient BEU/DMF solutions with (a) λex = 312 nm and (b) λex = 350 nm and their corresponding excitation spectra with (a) λem = 354 nm and (b) λem = 417 nm. Figure S9: photographs of gradient BEU/DMF solutions under (a) 312 nm UV and (b) 365 nm UV with Φc values (λex = 365 nm). Figure S10: emission spectra of 0.1 M BEU/DMF solutions with different λexs. Figure S11: prompt and delayed emission spectra of 10−5 M BEU/DMF solution at 77 K. (λex = 312 nm, td = 0.1 ms) Figure S12: lifetimes of p-RTP for (a) MBEU-1, (b) MBEU-2, and (c) CBEU crystals at different λems (λex = 312 nm). Figure S13: schematic illustration of the twisted molecular conformation of MBEU-1 crystals in the same layer. Figure S14: UV absorption of BEU, MBEU-1, MBEU-2, and CBEU crystals. Figure S15: (a) electron density distributions of HOMO and LUMO of MBEU-1 monomer and dimers. (b) Energy levels of MBEU-1 monomer and dimers. Figure S16: (a) electron density distributions of HOMO and LUMO of MBEU-2 monomer and dim [file 9757460.f1.zip › Supporting Information (Clean Version).pdf]

# Time-Dependent Afterglow from a Single Component Organic Luminogen

Tianjia Yang†, Yunzhong Wang†, Jixuan Duan, Shuangyu Wei, Saixing Tang, and Wang Zhang Yuan*

School of Chemistry and Chemical Engineering, Frontiers Science Center for Transformative Molecules, Shanghai Key Lab of Electrical Insulation and Thermal Aging, Shanghai Jiao Tong University, Shanghai 200240, China.
*E-mail: wzhyuan@sjtu.edu.cn

† These authors contributed equally to this work.

## Experimental Section

**Materials.** Benzoyleneurea (BEU, >99.5%) was purchased from Shanghai Adamas Reagent Co., Ltd. 6-methylquinazoline-2,4(1H,3H)-dione (MBEU-1, >98.0%), 8-methylquinazoline-2,4(1H,3H)-dione (MBEU-2, >98.0%), and 6-chloroquinazoline-2,4(1H,3H)-dione (CBEU, >99.0%) were purchased from Shanghai Bide Pharmatech Co., Ltd. N,N-Dimethylformamide (DMF, 99.9%) and tetrahydrofuran (THF, 99.9%) were purchased from J&K Scientific Co., Ltd. Pure water was purchased from Hangzhou Wahaha Co., Ltd. DMSO-$d_6$ was purchased from Cambridge Isotope Laboratories, Inc.

**Instrumentation.** $^1$H and $^{13}$C nuclear magnetic resonance (NMR) spectra were obtained from a Bruker DRX 500 NMR spectrometer (Germany) at room temperature. Prompt and delayed emission spectra, quantum efficiencies, as well as lifetime profiles of crystal samples were recorded on an Edinburgh FLS1000 photoluminescence spectrometer with a PMT900 detector. Excitation and emission spectra of solutions were determined at room temperature on a PerkinElmer LS55 luminescence spectrometer. Absorption spectra of solids were measured on PerkinElmer Lambda 750s spectrometer. All photographs and videos were taken by a mirrorless camera (Sony α7sII, Japan). Single crystal structures were characterized on a Bruker D8 VENTURE X-ray diffractometer with Cu-Kα X-ray source radiation (λ = 1.54184 Å) in the ω scan mode. High performance liquid chromatography (HPLC) was condcuted on an Agilent 1260 HPLC with a UV detector. The preparation of BEU solids was conducted on the a Waters Prep 150 LC.

**Sample Purification.** The powders were dissolved in mixed solvent of 50:50 THF/water (v/v) at 50 °C. The hot saturated solutions were then cooled down to 4 °C to yield colorless crystals.

**Single crystal cultivation.** Single crystals suitable for SC-XRD were obtained by slow evaporation of corresponding THF solutions. The collected crystals were dried under vacuum at 40 °C overnight before spectra measurements.

**High Performance Liquid Chromatography.** The solids were first dissolved in mixed solvent of 50:50 DMF/$CH_3OH$ (v/v) with a concentration of 5 mg/mL. Then the solutions were filtered and centrifuged

to yield solutions ready for HPLC injection. The eluent was composed of different ratios of $CH_3OH$ and $H_2O$ with a gradient elution program.

**Theorytical Calculation.** The molecular models for calculation were extracted from corresponding single-crystal structures without further geometry optimization. TD-DFT calculations at B3LYP/6-31G(d,p) level were conducted on a Gaussian 16 (version A.03) program[S1].

**Note.** Unless specified, all measurements were conducted at ambient conditions.

**Figure S1.** $^1$H NMR spectrum of (a) BEU, (b) MBEU-1, (c) MBEU-2 and (d) CBEU in DMSO-$d_6$.

**Figure S2.** $^{13}$C NMR spectrum of (a) BEU, (b) MBEU-1, (c) MBEU-2 and (d) CBEU in DMSO-$d_6$.

**Figure S3.** HPLC diagrams of the four compounds.

**Figure S4.** Photographs of BEU solid purified by HPLC under 312 nm UV excitation or after ceasing the irridiation.

**Table S1.** Single crystal data of BEU, MBEU-1, MBEU-2, and CBEU.

**Figure S5.** Ns-lifetime decay of BEU crystals at (a) 365 nm and (b) 428 nm.

**Table S2.** RTP lifetimes and their value proportions of BEU crystals.

**Figure S6.** (a) Delayed emission spectra ($t_d$ = 1ms) of BEU crystals at 77 K with different $\lambda_{ex}$s. (b) Lifetime decays of BEU crystals at different $\lambda_{em}$s at 77 K ($\lambda_{ex}$ = 340 nm).

**Table S3.** Cryogenic phosphorescence lifetimes and their value proportions of BEU crystals.

**Figure S7.** (a) Delayed emission spectra of BEU with different delayed times ($\lambda_{ex}$ = 340 nm) at 77 K. (b) CIE coordinates of the recorded spectra.

**Figure S8.** Emission spectra of gradient BEU/DMF solutions with (a) $\lambda_{ex}$ = 312 nm and (b) $\lambda_{ex}$ = 350 nm and their corresponding excitation spectra with (a) $\lambda_{em}$ = 354 nm and (b) $\lambda_{em}$ = 417 nm.

**Figure S9.** Photographs of gradient BEU/DMF solutions under (a) 312 nm UV and (b) 365 nm UV with $\Phi_c$ values ($\lambda_{ex}$ = 365 nm).

**Figure S10.** Emission spectra of 0.1 M BEU/DMF solutions with different $\lambda_{ex}$s.

**Figure S11.** Prompt and delayed emission spectra of $10^{-5}$ M BEU/DMF solution at 77 K. ($\lambda_{ex}$ = 312 nm, $t_d$ = 0.1 ms)

**Figure S12.** Lifetimes of p-RTP for (a) MBEU-1, (b) MBEU-2, and (c) CBEU crystals at different $\lambda_{em}$s ($\lambda_{ex}$ = 312 nm)

**Figure S13.** Schematic illustration of the twisted molecular conformation of MBEU-1 crystals in the same layer.

**Figure S14.** UV absorption of BEU, MBEU-1, MBEU-2, and CBEU crystals.

**Figure S15.** (a) Electron density distributions of HOMO and LUMO of MBEU-1 monomer and dimers. (b) Energy levels of MBEU-1 monomer and dimers.

**Figure S16.** (a) Electron density distributions of HOMO and LUMO of MBEU-2 monomer and dimers. (b) Energy levels of MBEU-2 monomer and dimers.

**Figure S17.** (a) Electron density distributions of HOMO and LUMO of CBEU monomer and dimers. (b) Energy levels of CBEU monomer and dimers.

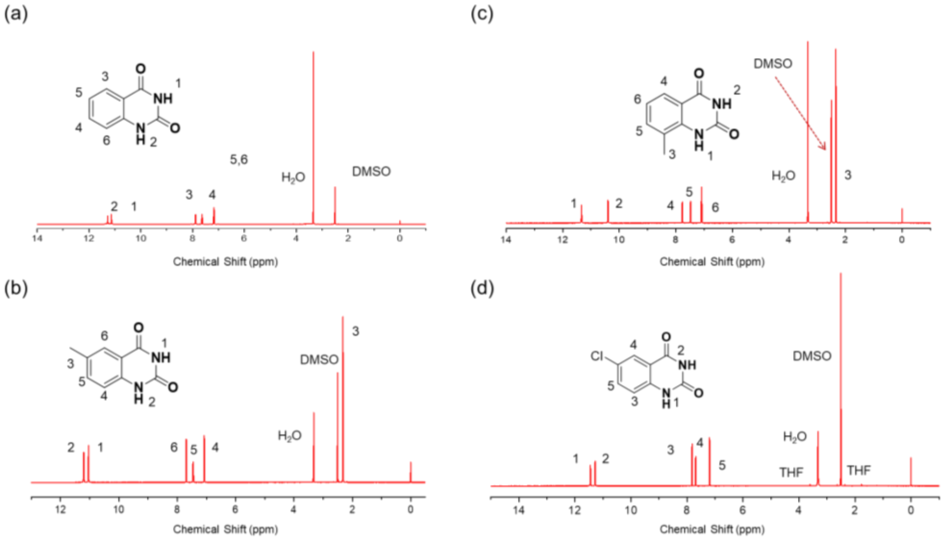


**Figure S1.** $^{1}$H NMR spectrum of (a) BEU, (b) MBEU-1, (c) MBEU-2 and (d) CBEU in DMSO-$d_6$.

BEU: $^{1}$H NMR (500 MHz, DMSO-*d6*) δ 11.28 (s, 1H), 11.14 (s, 1H), 7.89 (dd, J = 7.9, 1.4 Hz, 1H), 7.64 (ddd, J = 8.5, 7.4, 1.6 Hz, 1H), 7.18 (ddd, J = 9.6, 7.7, 1.6 Hz, 2H).

MBEU-1: $^{1}$H NMR (500 MHz, DMSO-$d_6$) δ 11.20 (s, 1H), 11.04 (d, *J* = 2.0 Hz, 1H), 7.69 (s, 1H), 7.46 (d, *J* = 7.9 Hz, 1H), 7.07 (d, *J* = 8.3 Hz, 1H), 2.32 (s, 3H).

MBEU-2: $^{1}$H NMR (500 MHz, DMSO-$d_6$) δ 11.33 (s, 1H), 10.40 (s, 1H), 7.77 (dd, *J* = 7.9, 1.4 Hz, 1H), 7.48 (dt, *J* = 7.4, 1.1 Hz, 1H), 7.09 (t, *J* = 7.6 Hz, 1H), 2.34 (s, 3H).

CBEU: $^{1}$H NMR (500 MHz, DMSO-$d_6$) δ 11.45 (s, 1H), 11.27 (d, *J* = 1.9 Hz, 1H), 7.82 (d, *J* = 2.5 Hz, 1H), 7.69 (dd, *J* = 8.7, 2.5 Hz, 1H), 7.19 (d, *J* = 8.7 Hz, 1H).

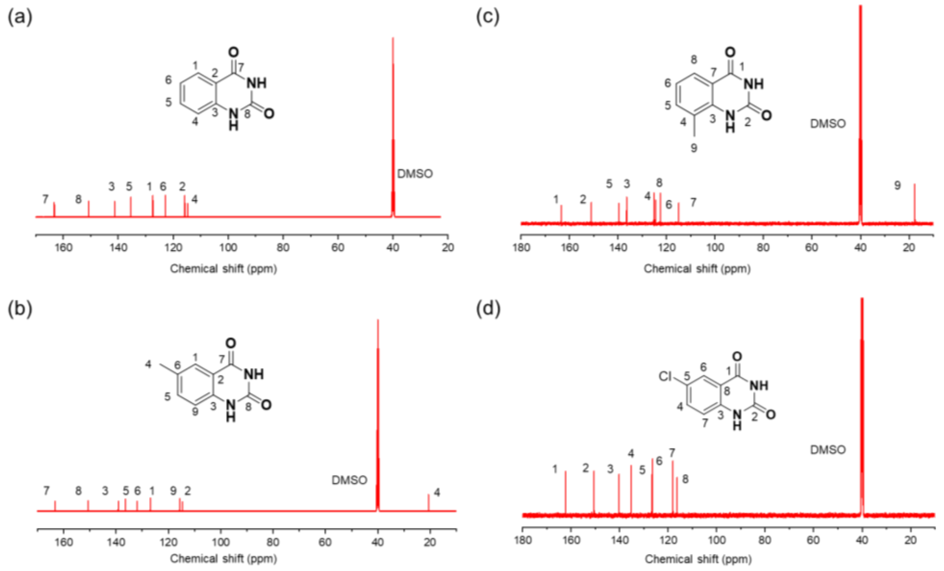


**Figure S2.** $^{13}$C NMR spectrum of (a) BEU, (b) MBEU-1, (c) MBEU-2 and (d) CBEU in DMSO-$d_6$.

BEU: $^{13}$C NMR (126 MHz, DMSO-$d_6$) δ 163.30, 150.76, 141.33, 135.43, 127.42, 122.79, 115.78, 114.80.

MBEU-1: $^{13}$C NMR (126 MHz, DMSO-$d_6$) δ 163.29, 150.74, 139.16, 136.41, 131.99, 126.92, 115.74, 114.61, 20.67.

MBEU-2: $^{13}$C NMR (126 MHz, DMSO) δ 163.40, 150.95, 139.66, 136.43, 125.21, 124.55, 122.51, 114.97, 17.63.

CBEU: $^{13}$C NMR (126 MHz, DMSO-$d_6$) δ 162.30, 150.51, 140.20, 135.30, 126.76, 126.39, 118.00, 116.26.

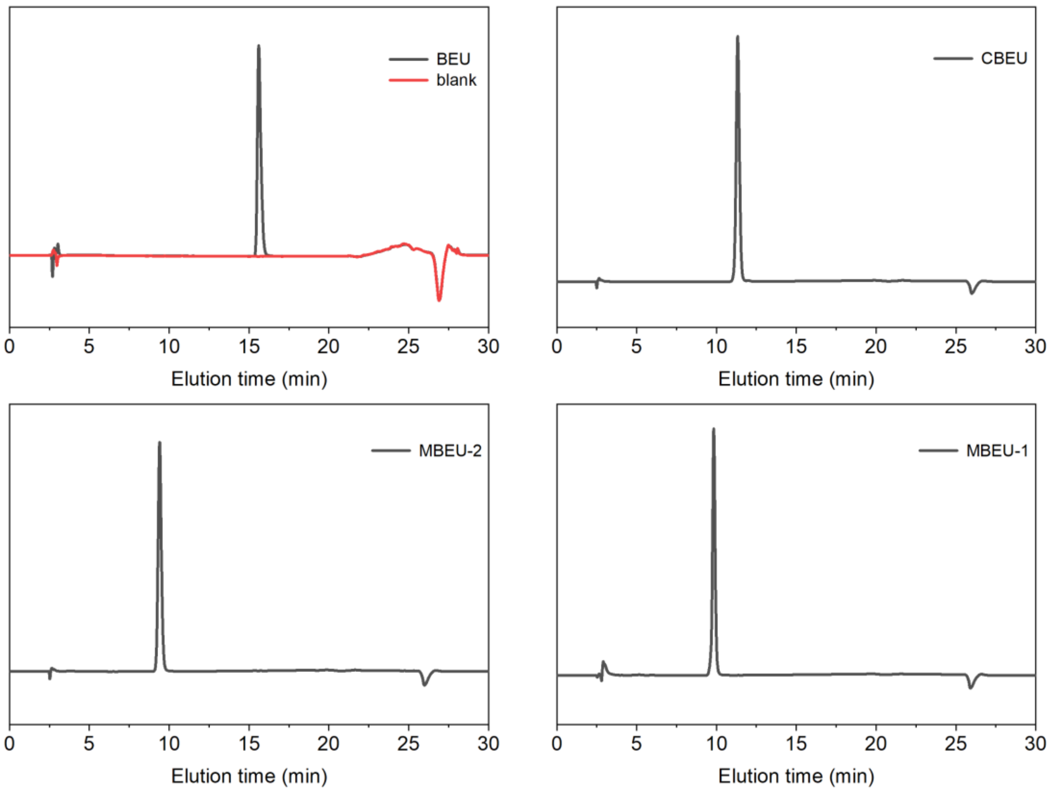


**Figure S3.** HPLC diagrams of the four compounds.

(Note: the peaks observed at around 2.5 min of elution time should be attributed to the signal for the solutions (typically DMF) which is also observed in the blank curve. The reversed peak at around 26 min should also be attributed to the solution background, which is also observed in the blank curve.)

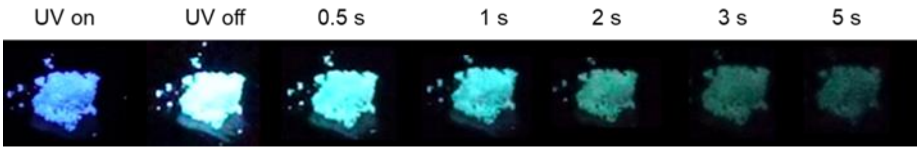


**Figure S4.** Photographs of BEU solid purified by HPLC under 312 nm UV excitation or after ceasing the irridiation.

**Table S1.** Single crystal data of BEU, MBEU-1, MBEU-2, and CBEU.[S2]

| | BEU | MBEU-1 | MBEU-2•$H_2O$ | CBEU |
|---|---|---|---|---|
| CCDC | 2079947 | 2079945 | 2079948 | 2079946 |
| Formula | $C_8H_6N_2O_2$ | $C_9H_8N_2O_2$ | $C_9H_{10}N_2O_3$ | $C_8H_5ClN_2O_2$ |
| Formula Weight | 162.15 | 176.17 | 194.19 | 196.59 |
| Wavelength (Å) | 1.54178 | 1.54178 | 1.54178 | 1.54178 |
| Space Group | P21/c | P21/n | P21/c | P21/c |
| Cell Length (Å) | a = 10.8541(5) | a = 15.3058(12) | a = 11.7154(6) | a = 14.0230(16) |

| | | | | |
|---|---|---|---|---|
| | b = 5.2696(3) | b = 5.0105(4) | b = 4.8996(3) | b = 3.8795(4) |
| | c = 12.6736(6) | c = 21.0179(16) | c = 16.6621(9) | c = 14.0230(16) |
| Cell Angle (°) | α = 90 | α = 90 | α = 90 | α = 90 |
| | β = 90.598(2) | β = 94.78 | β = 110.58 | β = 94.26 |
| | γ = 90 | γ = 90 | γ = 90 | γ = 90 |
| Cell Volume (Å$^3$) | 714.74(6) | 1606.3(2) | 895.36(9) | 760.77(15) |
| *Z* | 4 | 8 | 4 | 4 |
| Density (g cm$^{-3}$) | 1.507 | 1.457 | 1.441 | 1.716 |
| F (000) | 336 | 736 | 408 | 400 |
| $h_{max}$, $k_{max}$, $l_{max}$ | 13, 6, 15 | 17, 5, 24 | 14, 5, 20 | 16, 4, 16 |
| $T_{min}$, $T_{max}$ | 0.659, 0.753 | 0.564, 0.753 | 0.596, 0.753 | 0.614, 0.753 |

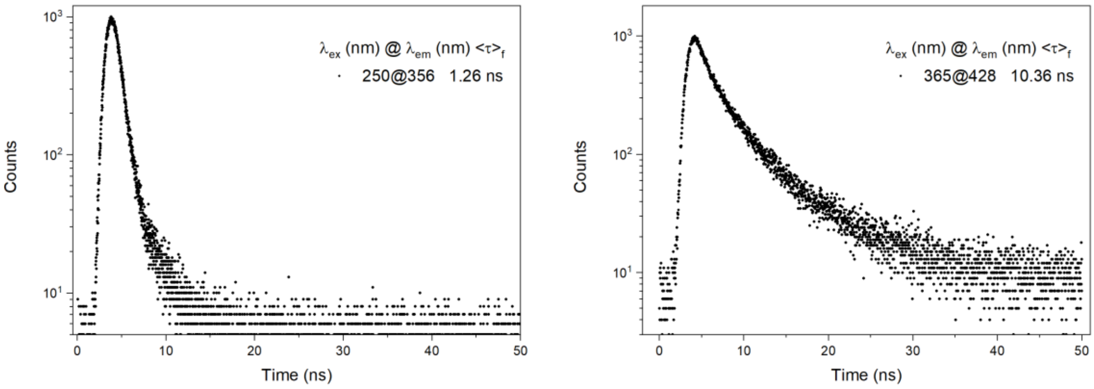


**Figure S5.** Ns-lifetime decay of BEU crystals at (a) 365 nm and (b) 428 nm.

**Table S2.** RTP lifetimes and their value proportions of BEU crystals.

| $\lambda_{ex}$ [nm] | $\lambda_{em}$ [nm] | $<\tau>_1$ [ms] | $<\tau>_2$ [ms] | $<\tau>_3$ [ms] | $B_1$ [%] | $B_2$ [%] | $B_3$ [%] | $\chi^2$ | $<\tau>$ [ms] |
|---|---|---|---|---|---|---|---|---|---|
| 312 | 434 | 56.00 | 206.24 | -- | 24.62 | 75.38 | -- | 1.320 | 169.25 |
| 312 | 500 | 26.81 | 181.29 | 729.95 | 11.53 | 65.02 | 23.45 | 1.38 | 292.14 |
| 340 | 434 | 199.86 | 369.48 | -- | 88.74 | 11.26 | -- | 1.02 | 218.96 |
| 340 | 500 | 64.57 | 222.79 | 754.44 | 7.47 | 78.90 | 13.63 | 0.95 | 283.44 |
| 365 | 434 | 67.97 | 225.99 | -- | 38.58 | 61.42 | -- | 1.22 | 139.06 |
| 365 | 500 | 42.43 | 165.40 | 811.24 | 18.25 | 62.84 | 18.91 | 1.25 | 264.98 |

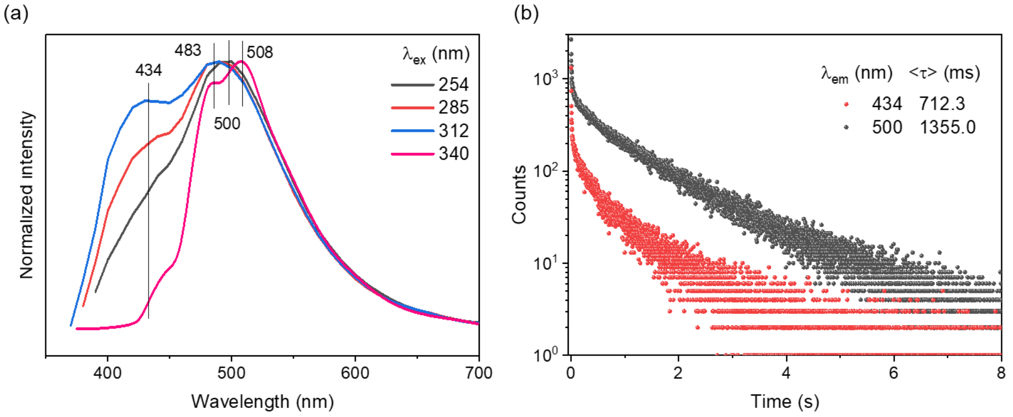


**Figure S6.** (a) Delayed emission spectra ($t_d$ = 1ms) of BEU crystals at 77 K with different $\lambda_{ex}$s. (b) Lifetime decays of BEU crystals at different $\lambda_{em}$s at 77 K ($\lambda_{ex}$ = 340 nm).

**Table S3.** Cryogenic phosphorescence lifetimes and their value proportions of BEU crystals at 77 K.

| $\lambda_{ex}$ [nm] | $\lambda_{em}$ [nm] | $<\tau>_1$ [ms] | $<\tau>_2$ [ms] | $<\tau>_3$ [ms] | $B_1$ [%] | $B_2$ [%] | $B_3$ [%] | $\chi^2$ | $<\tau>$ [ms] |
|---|---|---|---|---|---|---|---|---|---|
| 340 | 434 | 23.36 | 712.34 | -- | 11.38 | 88.62 | -- | 1.24 | 633.95 |
| 340 | 500 | 8.04 | 294.29 | 1354.95 | 2.65 | 15.81 | 81.54 | 1.189 | 1151.61 |

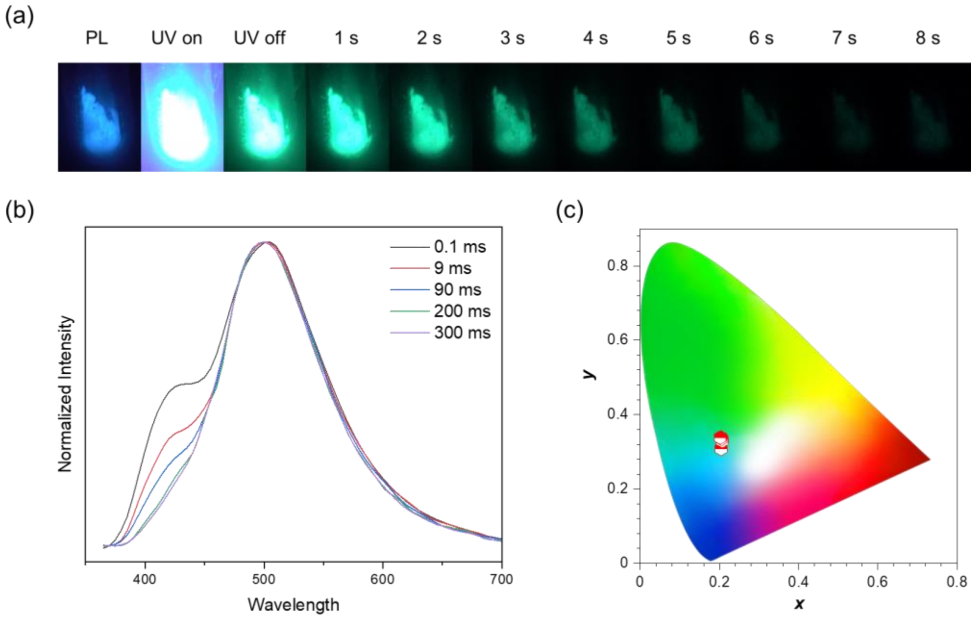


**Figure S7.** (a) Photographs of BEU single-crystals taken with 312 UV light or after ceasing the irradiation at different time points at 77 K. (b) Delayed emission spectra of BEU with different delayed times ($\lambda_{ex}$ = 340 nm) at 77 K. (c) CIE coordinates of the recorded spectra.

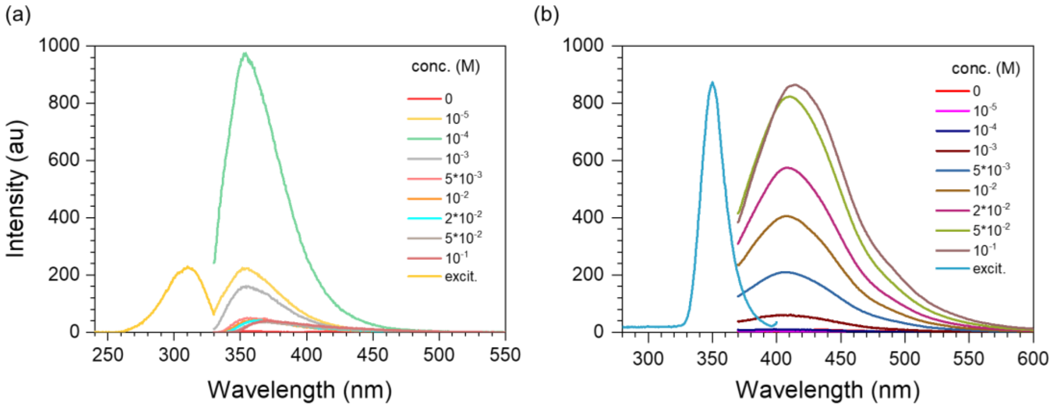


**Figure S8.** Emission spectra of gradient BEU/DMF solutions with (a) $\lambda_{ex}$ = 312 nm and (b) $\lambda_{ex}$ = 350 nm and their corresponding excitation spectra with (a) $\lambda_{em}$ = 354 nm and (b) $\lambda_{em}$ = 417 nm.

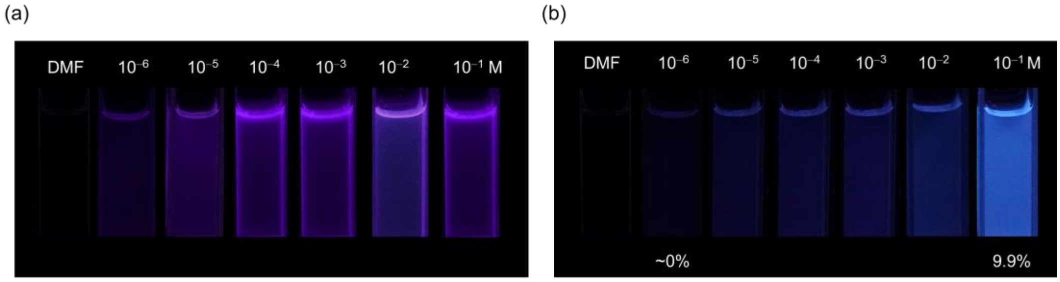


**Figure S9.** Photographs of gradient BEU/DMF solutions under (a) 312 nm UV and (b) 365 nm UV with $\Phi_c$ values ($\lambda_{ex}$ = 365 nm).

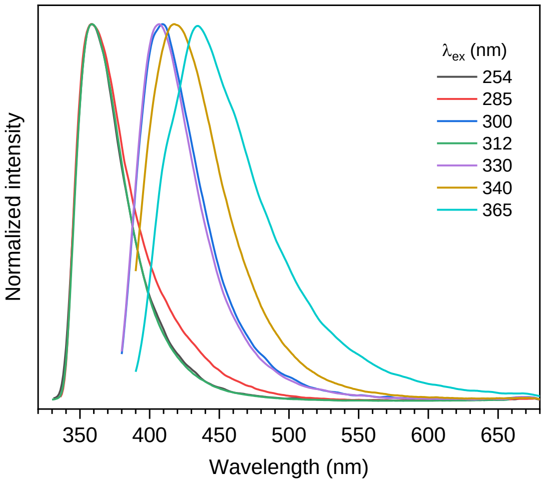


**Figure S10.** Emission spectra of 0.1 M BEU/DMF solutions with different $\lambda_{ex}$s.

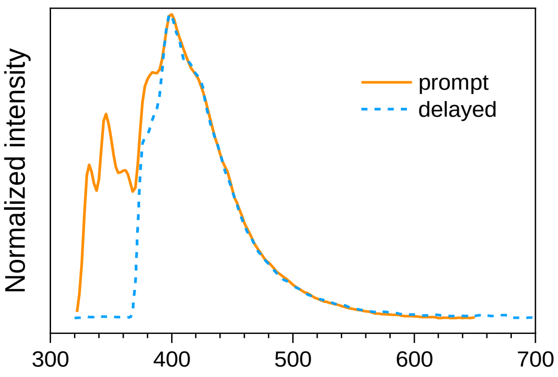


**Figure S11.** Prompt and delayed emission spectra of $10^{-5}$ M BEU/DMF solution at 77 K. ($\lambda_{ex}$ = 312 nm, $t_d$ = 0.1 ms)

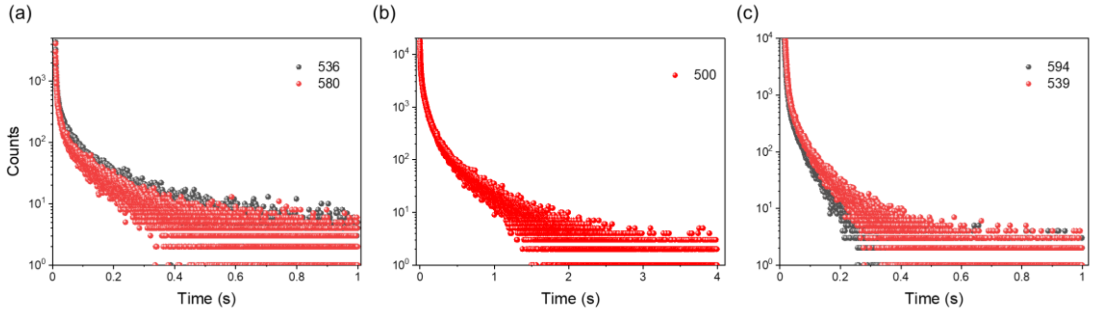


**Figure S12.** Lifetimes of p-RTP for (a) MBEU-1, (b) MBEU-2, and (c) CBEU crystals at different $\lambda_{em}$s ($\lambda_{ex}$ = 312 nm)

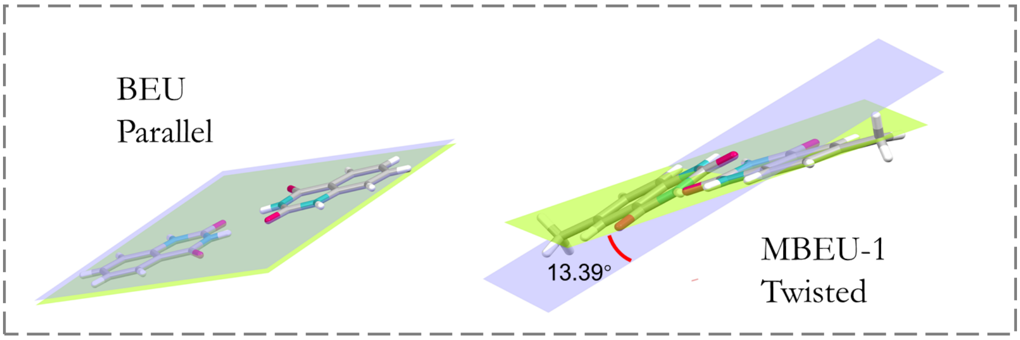


**Figure S13.** Schematic illustration of the twisted molecular conformation of MBEU-1 crystals in the same layer.

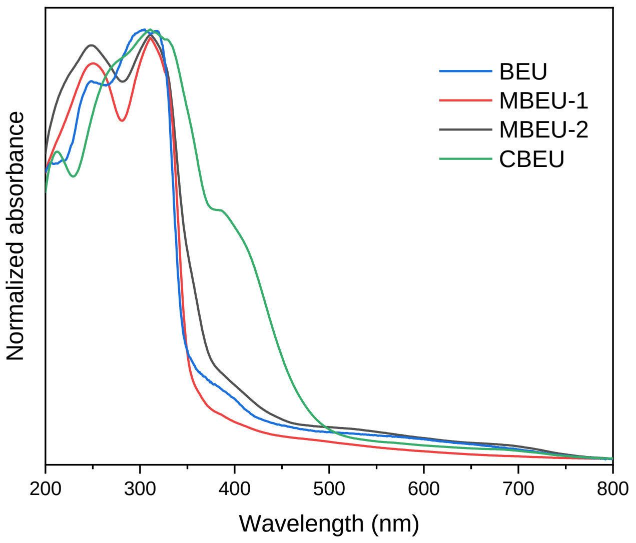


**Figure S14.** UV absorption of BEU, MBEU-1, MBEU-2, and CBEU crystals.

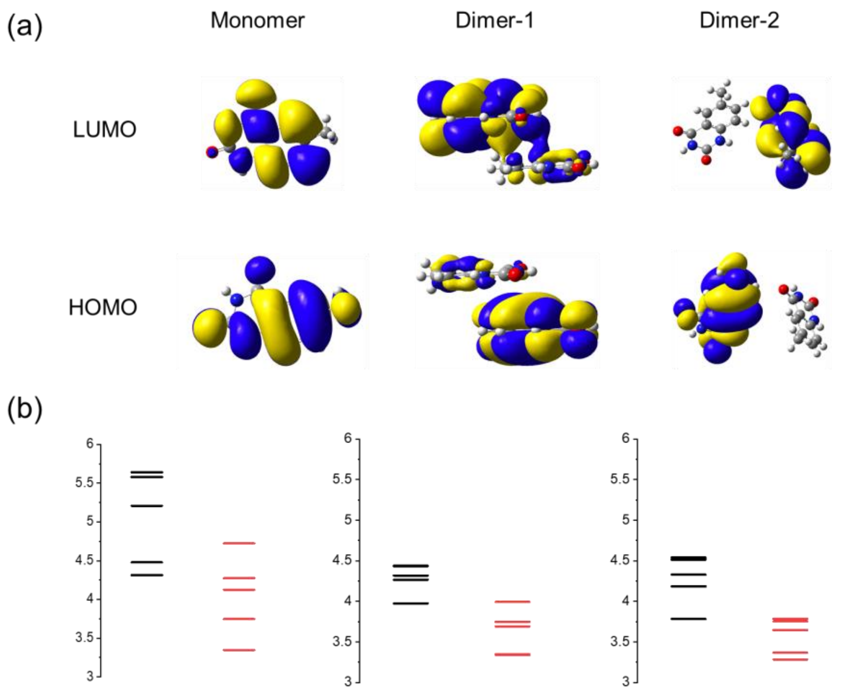


**Figure S15.** (a) Electron density distributions of HOMO and LUMO of MBEU-1 monomer and dimers. (b) Excited states energy levels of MBEU-1 monomer and dimers.

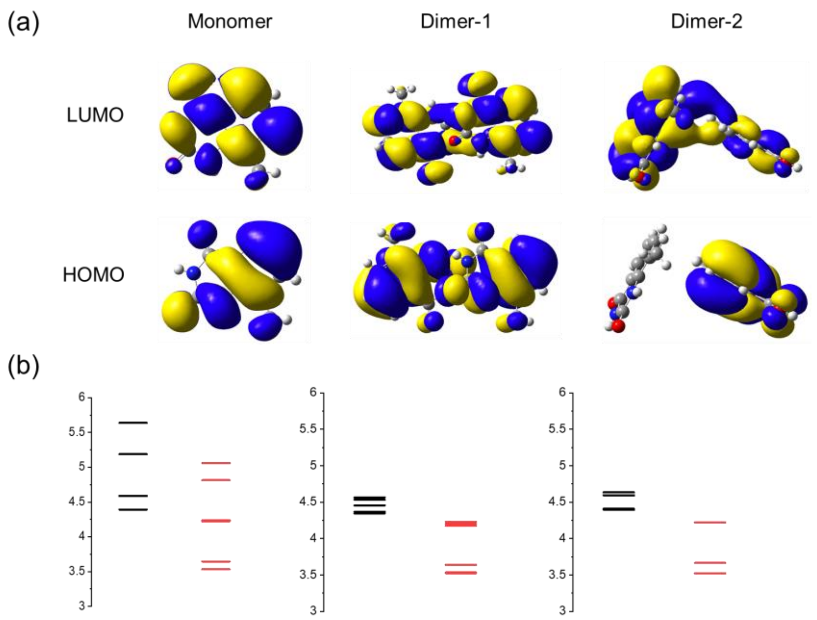


**Figure S16.** (a) Electron density distributions of HOMO and LUMO of MBEU-2 monomer and dimers. (b) Excited states energy levels of MBEU-2 monomer and dimers.

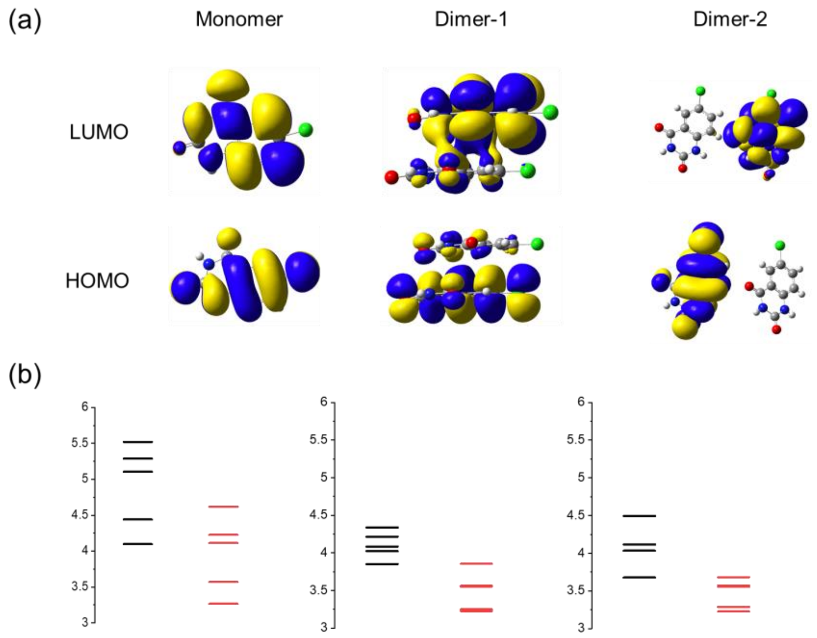


**Figure S17.** (a) Electron density distributions of HOMO and LUMO of CBEU monomer and dimers. (b) Excited states energy levels of CBEU monomer and dimers.

## References


[S1] Gaussian 16, Revision A.03, M. J. Frisch, G. W. Trucks, H. B. Schlegel, G. E. Scuseria, M. A. Robb, J. R. Cheeseman, G. Scalmani, V. Barone, G. A. Petersson, H. Nakatsuji, X. Li, M. Caricato, A. V. Marenich, J. Bloino, B. G. Janesko, R. Gomperts, B. Mennucci, H. P. Hratchian,

J. V. Ortiz, A. F. Izmaylov, J. L. Sonnenberg, D. Williams-Young, F. Ding, F. Lipparini, F. Egidi, J. Goings, B. Peng, A. Petrone, T. Henderson, D. Ranasinghe, V. G. Zakrzewski, J. Gao, N. Rega, G. Zheng, W. Liang, M. Hada, M. Ehara, K. Toyota, R. Fukuda, J. Hasegawa, M. Ishida, T. Nakajima, Y. Honda, O. Kitao, H. Nakai, T. Vreven, K. Throssell, J. A. Montgomery, Jr., J. E. Peralta, F. Ogliaro, M. J. Bearpark, J. J. Heyd, E. N. Brothers, K. N. Kudin, V. N. Staroverov, T. A. Keith, R. Kobayashi, J. Normand, K. Raghavachari, A. P. Rendell, J. C. Burant, S. S. Iyengar, J. Tomasi, M. Cossi, J. M. Millam, M. Klene, C. Adamo, R. Cammi, J. W. Ochterski, R. L. Martin, K. Morokuma, O. Farkas, J. B. Foresman, and D. J. Fox, Gaussian, Inc., Wallingford CT, 2016.

[S2] CCDC 2079947 (BEU), 2079945 (MBEU-1), 2079948 (MBEU-2), and 2079946 (CBEU) contain the supplementary crystallographic data. These data can be obtained free of charge from The Cambridge Crystallographic Data Centre.
